# Bioavailable central nervous system disease-modifying therapies for multiple sclerosis

**DOI:** 10.3389/fimmu.2023.1290666

**Published:** 2023-11-29

**Authors:** Hans-Peter Hartung, Bruce A.C. Cree, Michael Barnett, Sven G. Meuth, Amit Bar-Or, Lawrence Steinman

**Affiliations:** ^1^ Department of Neurology, Medical Faculty, Heinrich-Heine University, Düsseldorf, Germany; ^2^ Brain and Mind Centre, University of Sydney, Sydney, NSW, Australia; ^3^ Department of Neurology, Medical University of Vienna, Vienna, Austria; ^4^ Department of Neurology, Palacký University Olomouc, Olomouc, Czechia; ^5^ Weill Institute for Neurosciences, Department of Neurology, University of California San Francisco, San Francisco, CA, United States; ^6^ Center for Neuroinflammation and Experimental Therapeutics, Department of Neurology, Perelman School of Medicine, University of Pennsylvania, Philadelphia, PA, United States; ^7^ Department of Neurology and Neurological Sciences, Beckman Center for Molecular Medicine, Stanford University Medical Center, Stanford, CA, United States

**Keywords:** sphingosine 1 phosphate receptor modulators, multiple sclerosis, ozanimod, cladribine, ponesimod, siponimod, fingolimod hydrochloride, central nervous system

## Abstract

Disease-modifying therapies for relapsing multiple sclerosis reduce relapse rates by suppressing peripheral immune cells but have limited efficacy in progressive forms of the disease where cells in the central nervous system play a critical role. To our knowledge, alemtuzumab, fumarates (dimethyl, diroximel, and monomethyl), glatiramer acetates, interferons, mitoxantrone, natalizumab, ocrelizumab, ofatumumab, and teriflunomide are either limited to the periphery or insufficiently studied to confirm direct central nervous system effects in participants with multiple sclerosis. In contrast, cladribine and sphingosine 1-phosphate receptor modulators (fingolimod, ozanimod, ponesimod, and siponimod) are central nervous system-penetrant and could have beneficial direct central nervous system properties.

## Introduction

**Figure 1:**
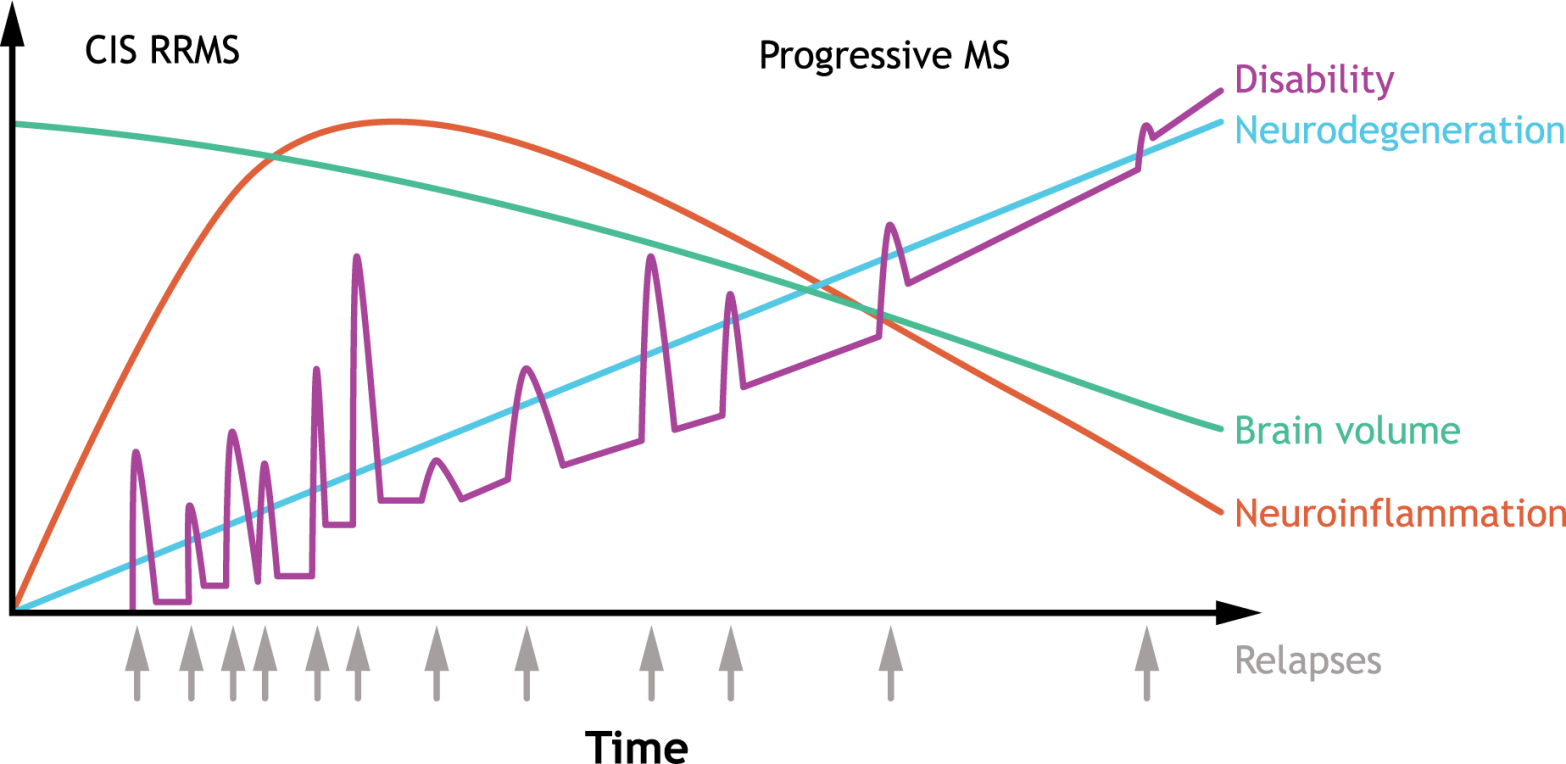
Typical clinical course of MS. The clinical course of MS is heterogenous, but RRMS is the most common subtype characterized by an initial episode of neurological symptoms (CIS) followed by periods of remissions and relapses. Some patients have an initial diagnosis of primary progressive MS, which is associated with progressive decline. Over time, improvement during remissions wane, and disability accumulates, with most patients developing secondary progressive MS. In secondary progressive MS, neurodegeneration is accompanied with a decline in brain volume. CIS, clinically isolated syndrome; MS, multiple sclerosis; RRMS, relapsing-remitting multiple sclerosis. Source: Adapted (colors revised, text edited/moved, and content removed) from Håkansson et al. (2). under Creative Commons Attribution NonCommercial 4.0 International License (https://creativecommons.org/licenses/by-nc/4.0/).

As of 2020, approximately 2.8 million people were recorded to have multiple sclerosis (MS) worldwide (global prevalence: 35.9 [95% confidence interval, 35.87–35.95] per 100,000 persons), with women being twice as likely as men to develop MS (1), highlighting the need for effective disease-modifying therapies (DMTs). The typical progression of MS is shown in **Figure 1** (2). Current treatment strategies aim at reducing relapse risk, decreasing the formation of new brain lesions, and slowing disability progression (3, 4). Most currently available DMTs are immunomodulatory or immunosuppressive agents that act predominantly on peripheral immune responses (3, 4). While immunotherapies reduce relapse activity, most therapies for relapsing MS (RMS) have limited efficacy in treating progressive forms of MS, and targeting peripheral inflammation is not sufficient to slow disease progression (5).

As MS progresses, resident central nervous system (CNS) cells play a more dominant, critical role in MS pathogenesis; thus, the ability of DMTs to cross the blood-brain barrier (BBB) and directly target sites of inflammation, demyelination, and neuroaxonal damage could have additional benefits over therapies that are limited to the periphery (6, 7). For DMTs to be effective in progressive MS, they must also target chronic CNS inflammation (5). The purpose of this narrative review is to provide an overview of therapies currently approved by the US Food and Drug Administration (FDA) and European Medicines Agency (EMA) for MS treatment that penetrate the CNS and to elaborate on future perspectives in the MS therapeutic landscape.

## Methods

DMTs approved by the FDA and EMA as of February 2023 were included in this review. Pivotal phase 3 clinical trials in adults with RMS or progressive MS subtypes were assessed for alemtuzumab (CARE-MS I and CARE-MS II) (8, 9), dimethyl fumarate (CONFIRM and DEFINE) (10, 11), glatiramer acetate (Copolymer 1 Multiple Sclerosis Study Group) (12), interferons (IFN) (IFNB Multiple Sclerosis Study Group, Multiple Sclerosis Collaborative Research Group, European Study Group on IFN β-1b in Secondary Progressive MS, CHAMPS, EVIDENCE, BENEFIT, and ADVANCE) (13–20), mitoxantrone (MIMS) (21), natalizumab (AFFIRM) (22), ocrelizumab (OPERA I, OPERA II, and ORATORIO) (23, 24), ofatumumab (ASCLEPIOS I and ASCLEPIOS II) (25), teriflunomide (TEMSO and TOWER) (26, 27), cladribine (CLARITY) (28), fingolimod (FREEDOMS, FREEDOMS II, and TRANSFORMS) (29–31), ozanimod (SUNBEAM and RADIANCE) (32, 33), ponesimod (OPTIMUM) (34), and siponimod (EXPAND) (35).

Given the relatively recent FDA and EMA approvals of a subset of DMTs assessed here (e.g., ofatumumab, ozanimod, ponesimod), additional phase 3 clinical trials, *post hoc* analyses of phase 3 clinical trials, or open-label extension trials that enrolled phase 3 participants were included if relevant data were not reported in the pivotal phase 3 clinical trial publications or there were no significant differences in any measures of brain volume, cognition, or disability against a comparator in ≥1 pivotal study. Other relevant references were retrieved from October 2021 to July 2022 by searching PubMed for the generic name of each DMT combined with each of the following search terms individually: astrocyte, brain volume/atrophy, evoked potential latency, magnetic transfer ratio, microglia, multiple sclerosis, myelination, myelin water fraction, neuron, oligodendrocyte, oligodendrocyte progenitor/precursor cells, phase 3, remyelination, slowly expanding lesions (SELs), and smoldering lesions. All references were included at the authors’ discretion.

DMTs were categorized as direct CNS agents based on mechanism of action, ability to traverse the BBB, and compilation of data from the literature search (positive phase 3 clinical trial data for ≥2 measures of neurodegeneration that a direct-acting DMT would be expected to affect [brain volume, cognition, and/or disability]). Assessments of remyelination, most notably magnetic transfer ratio (MTR)/myelin water fraction (MWF) (36), were also evaluated. Ultimately, a DMT was considered limited to the periphery based on data suggesting limited ability or inability to cross the BBB, unavailable or nonsignificant phase 3 clinical trial data for the clinical outcomes assessed during the development of this narrative review, and the authors’ expert opinion.

## Observations

### Pathophysiology

**Figure 2:**
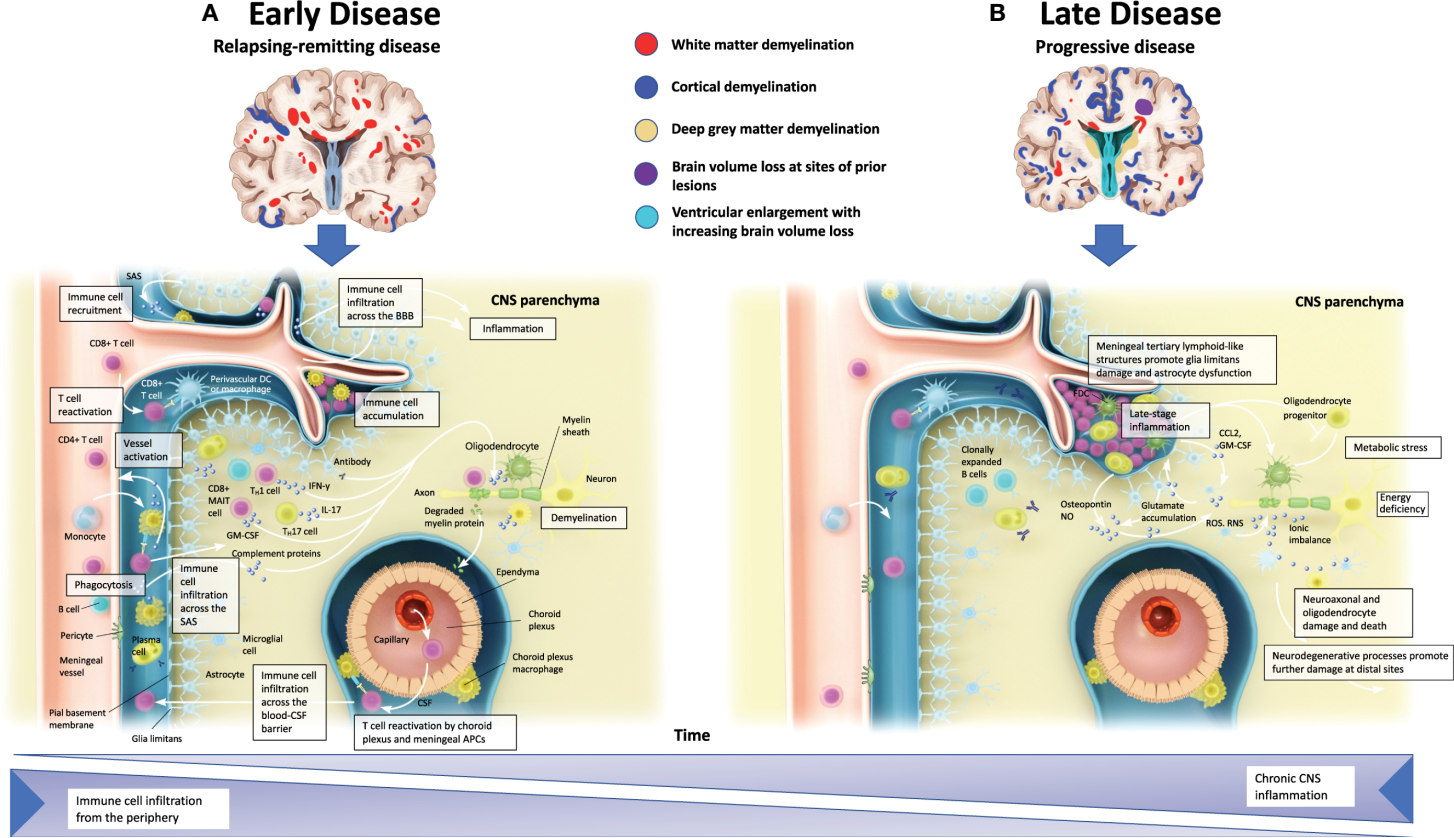
Pathophysiology of multiple sclerosis. **(A)** In the early stages of MS, relapses coincide CNS inflammation and demyelination that are typically discernible as white matter lesions via MRI. Peripheral immune cell infiltration can occur from meningeal blood vessels (across the BBB), the subarachnoid space, or the choroid plexus (across the blood-CSF barrier). These infiltrates may then accumulate in perivascular spaces and enter the CNS parenchyma, and along with activated CNS-resident microglia and astrocytes, promote demyelination and oligodendrocyte and neuroaxonal injury through direct contact-dependent mechanisms or via soluble inflammatory and neurotoxic mediators. **(B)** In the later stages of MS, progressive neurological decline is accompanied by CNS atrophy. Immune cell infiltration is dampened, but CNS inflammation persists. Meningeal tertiary lymphoid-like structures can also contribute to late-stage inflammation in SPMS. Irrespective of MS subtype, CNS-resident innate immune cells may contribute to chronic inflammation. Astrocytes produce ligands and factors that can promote microglial recruitment and activation while also preventing remyelination at the sites of neuroaxonal injury by inhibiting progenitor cell development into mature oligodendrocytes. APC, antigen-presenting cell; BBB, blood-brain barrier; CCL, chemokine ligand; CNS, central nervous system; CSF, cerebrospinal fluid; FDC, follicular dendritic cell; GM, grey matter; IFN-y, interferon gamma; IL, interleukin; MAIT, mucosal-associated invariant T cell; MRI, magnetic resonance imaging; MS, multiple sclerosis; NO, nitric oxide; RNS, reactive nitrogen species; ROS, reactive oxygen species; SPMS, secondary progressive multiple sclerosis; TH, T helper. Adapted from Dendrou et al. (38).

The BBB normally controls immune cell infiltrates in the CNS by limiting cell entry to only cell subsets required for immune surveillance (37). During periods of neuroinflammation, the BBB is impaired and immune cell entry into the CNS becomes less regulated (37). Early RMS pathophysiology is characterized by BBB injury, influx of peripheral immune cells into the CNS, and the development of focal inflammatory demyelinating lesions in grey and white matter [**Figure 2A** (38)] (4, 39). Breakdown of the BBB in patients with MS may therefore facilitate pro​inflammatory cell migration into the CNS (40). During relapses, which are most prominent early in MS, peripheral innate and adaptive immune cells, such as CD4+ and CD8+ T cells, B cells, and myeloid cells, accumulate in the perivascular space and infiltrate the CNS parenchyma (4, 41–44).

Peripheral immune cells, resident activated microglia, and astrocytes likely contribute to oligodendrocyte injury, demyelination, and neuroaxonal injury mediated by the secretion of soluble factors and cell contact–dependent mechanisms (4). While the formation of MS lesions is associated with acute axonal injury, subsequent lesion-related Wallerian, retrograde, and neuroaxonal degeneration contribute to the ongoing loss of brain tissue (45, 46).

As the disease progresses, compartmentalized inflammatory mechanisms that predominantly involve microglia, B cells, and other innate cells of the CNS become more prominent, leading to neurodegeneration, CNS atrophy, and disability worsening [**Figure 2B** (38, 47–51)]. The influx of immune cells into the CNS is dampened, but CNS inflammation persists from resident innate immune cells contributing to a chronic, localized inflammatory response (38). Other mechanisms that contribute to progressive tissue injury and the symptoms associated with progressive forms of the disease may include neurodegeneration resulting from neuroaxonal, astrocyte, and oligodendrocyte damage (4, 52). This tissue injury is evident at the edge of SELs, where “slow-burning” inflammation contributes to increased lesion burden, brain atrophy, cognition, and disability worsening (53, 54). In progressive MS, the BBB is an essential barrier for DMTs to overcome target sites of CNS-compartmentalized inflammation, demyelination, and neuroaxonal damage (6, 7). Clinical efficacy on measures of cognition, brain volume, and disability may therefore indicate direct CNS effects (55–57).

Although small, lipophilic molecules cross the BBB through passive diffusion, large molecules require membrane transport proteins to cross the BBB (58). For large molecule MS therapies that are unable to cross the BBB, it is proposed that effects on peripheral immune cells indirectly affect CNS-compartmentalized processes, potentially through changing the function or circulating concentration of immune cells that would typically access the CNS (59); thus, depending on target cells and the size/lipophilic nature of the compound, MS therapies may have CNS and peripheral effects.

### Direct CNS and peripheral effects of DMTs

The evidence presented in **Supplementary Table 1** for alemtuzumab; dimethyl, diroximel, and monomethyl fumarate; interferons; glatiramer acetate; mitoxantrone; natalizumab; ocrelizumab; ofatumumab; and teriflunomide suggests that the function of these DMTs may be limited to the periphery or that they were insufficiently studied to confirm direct CNS effects. Evidence for cladribine, fingolimod, ozanimod, ponesimod, and siponimod suggests their direct CNS effects (**Supplementary Table 2**); therefore, these DMTs are the focus of this review.

### DNA synthesis inhibitor

#### Cladribine

Cladribine is indicated for the treatment of adults with RMS, including relapsing-remitting MS (RRMS) and active secondary progressive MS (SPMS) in the US and for adults with highly active RMS in the European Union (EU) (60, 61). Cladribine is an adenosine analog prodrug thought to function by causing cytotoxic effects on B and T cells through the impairment of DNA synthesis resulting in lymphocyte depletion (60). Intermittent therapy with cladribine can induce long-term remission of MS that is sustained without ongoing treatment (28). During treatment-free periods, the immune system repopulates and regains the ability to respond to infections without commensurate return of MS disease activity; thus, cladribine is considered an immune reconstitution therapy (62, 63).

As a small molecule, cladribine crosses the BBB in humans (64, 65), with penetration of the CNS observed in children with acute leukemia (64). An *in vitro* study found cladribine treatment to significantly reduce the granularity, phagocytotic ability, and motility of lipopolysaccharide-stimulated microglia at concentrations that putatively overlap with those found in the cerebrospinal fluid (CSF) of humans (0.1 µM–1 µM) (66). Other preclinical studies confirmed that cladribine inhibits microglial proliferation and proinflammatory cytokine release and induces microglia apoptosis (67, 68).

In a phase 3, randomized, placebo-controlled trial, cladribine-treated participants with RMS had a lower risk of clinical relapse and disability progression and had suppression of magnetic resonance imaging (MRI) brain lesions compared with placebo over 96 weeks (28). A *post hoc* analysis found that cladribine treatment resulted in less annualized brain atrophy over 2 years compared with placebo-treated participants (69). This reduction in brain atrophy was closely associated with a lower risk of disability progression, suggesting that treatment with cladribine can target neurodegeneration in patients with RMS (69). An observational study also found that cladribine suppresses the intrathecal humoral response, as observed by the disappearance of oligoclonal banding from the CSF of participants with RMS, an effect that was associated with milder neurological disability after 10 years of follow-up (70). To our knowledge, phase 3 clinical trials assessing cognition and studies utilizing MTR/MWF techniques are not published for cladribine.

### S1P receptor modulators

#### Fingolimod

Fingolimod is indicated for the treatment of patients 10 years of age and older with RMS, including clinically isolated syndrome (CIS), RRMS, and active SPMS, in the US and for patients 10 years of age and older with highly active RMS in patients with active disease despite treatment with at least one other DMT or rapidly evolving severe RMS in the EU (71, 72). Fingolimod is a prodrug metabolized by sphingosine kinase to the active metabolite fingolimod-phosphate, which is a sphingosine-1 phosphate (S1P) receptor modulator that binds with high affinity to S1P_1_,_3_,_4_,_5_ and blocks the capacity of lymphocytes to egress from lymph nodes, reducing the number of lymphocytes in peripheral blood (71). The full mechanism by which fingolimod exerts therapeutic effects in MS is unknown but may involve lymphocyte sequestration within lymphatic tissue thereby limiting lymphocyte migration into the CNS (71).

The lipophilic properties of fingolimod allow for CNS penetration (73). In experimental autoimmune encephalomyelitis (EAE) rodent models, fingolimod crossed the BBB, accumulated in the white matter of the CNS along the myelin sheath, and reduced S1P_1_ signaling from astrocytes (73, 74). Following intravenous injection in humans, a radiolabeled fingolimod analog entered the brain, with uptake increasing up to 26 hours after administration (75). An *in vivo* study found fingolimod improves neurological functional recovery in an EAE mouse model and promotes oligodendrocyte precursor cell proliferation and differentiation (76). Additional *in vitro* studies observed ameliorated pathological effectors associated with the activation of microglia, ultimately leading to increased morphological markers of remyelination and direct neuronal effects (77, 78).

In phase 3, randomized, RMS clinical trials, treatment with fingolimod reduced annualized relapse rate (ARR), MRI brain lesion activity, and brain volume loss compared with placebo at 12 and 24 months or IFN β-1a at 12 months (29–31). *Post hoc* analysis of data pooled from the phase 3 FREEDOMS and FREEDOMS II trials found that early fingolimod treatment may offer long-term cognitive benefits in participants with RRMS, as determined via improvements in Paced Auditory Serial Addition Test 3 (PASAT-3) scores from baseline compared with placebo (79). Fingolimod also reduced the risk of 3- and 6-month confirmed disability progression (CDP) over a 24-month study period, with Expanded Disability Status Scale (EDSS) and Multiple Sclerosis Functional Composite (MSFC) z scores remaining stable or improving slightly at month 24 compared with placebo in participants with RMS (29); however, when compared with IFN β-1a treatment, there were no significant differences in time to disability progression or the proportion of participants with confirmed progression at 12 months (31). Subsequently, a *post hoc* analysis of data from TRANSFORMS, FREEDOMS, FREEDOMS II, and their extensions, found that participants taking continuous fingolimod were more likely to experience confirmed or sustained disability improvement over 8 years than those switching from IFN β-1a and were more likely to experience confirmed disability improvement than those switching from placebo, suggesting long-term benefit (80).

Fingolimod also enhanced tissue damage recovery assessed by MTR in lesions after 6 months and in normal-appearing white matter and grey matter after 2 years (81). In an investigator-driven, randomized, phase 2 trial, fingolimod treatment improved recovery from unilateral optic neuritis in participants with MS or CIS compared with IFN β-1b, further supporting effects on remyelination; however, these results require confirmation in multicenter phase 3 clinical trials (82).

#### Ozanimod

Ozanimod is indicated for the treatment of adults with RMS, including CIS, RRMS, and active SPMS in the US and for RRMS with active disease defined by clinical or imaging features in the EU, as well as ulcerative colitis in the US and EU (83, 84). Ozanimod is a selective S1P receptor modulator that binds with high affinity to S1P_1_ and S1P_5_ receptors. Ozanimod blocks lymphocyte egress from lymph nodes and reduces the number of lymphocytes in peripheral blood. Although its full mechanisms of action are unknown, ozanimod is proposed to affect MS through lymphocyte sequestration, thereby limiting lymphocyte migration into the CNS (84).

The ability of ozanimod to cross the BBB was observed preclinically, where the brain:blood ratio was 10:1 in mice following a single oral dose of ozanimod and 16:1 in rats following 5 days of once-daily dosing (85). Ex vivo treatment of EAE corticostriatal slices with ozanimod increased the mRNA expression of a marker of microglia activation while decreasing the expression of other inflammatory markers, and *in vitro* ozanimod treatment elicited potent protein kinase B (AKT) and extracellular-regulated kinase phosphorylation in human astrocytes (86, 87). In murine EAE, ozanimod reduced clinical scores at a dose that did not induce lymphopenia and elicited neuroprotective effects by reducing axonal breaks and improving functional capabilities following cuprizone-induced demyelination, each of these processes suggesting direct CNS effects (85, 88).

In phase 3, randomized, active comparator–controlled clinical trials, treatment with ozanimod 0.92 mg for up to 24 months reduced ARR, MRI brain lesions, and brain volume loss compared with IFN β-1a (32, 33). In addition, ozanimod treatment improved cognitive processing speed, as observed with greater Symbol Digit Modalities Test (SDMT) z scores, at month 12 compared with IFN β-1a in participants with RMS (32). Mean change in MSFC scores were similar across treatment groups from baseline to month 12 but were improved at month 24 with the ozanimod 0.46 mg dose compared with IFN β-1a (32, 33). Although the proportions of participants with CDP at 3 and 6 months were not significantly different between treatment groups in the phase 3 clinical trials (32, 33), interim analysis of a long-term, open-label extension trial demonstrated sustained control of disability progression with continuous ozanimod use (89). To our knowledge, studies utilizing MTR/MWF to assess the effects of ozanimod on demyelination are not published.

#### Ponesimod

Ponesimod is indicated for the treatment of adults with RMS, including CIS, RRMS, and active SPMS in the US and EU (90, 91). Ponesimod is an S1P receptor modulator that binds with high affinity exclusively to S1P_1_ and blocks the capacity of lymphocytes to egress from lymph nodes, reducing the number of lymphocytes in peripheral blood (90). Like the other S1P receptor modulators, ponesimod is proposed to act through lymphocyte sequestration and reduction of lymphocyte migration into the CNS (90).

Preclinical studies of ponesimod demonstrated dampened neuroinflammation through the attenuation of glial activation in murine EAE and neuroinflammatory responses in primary human astrocytes (92, 93). In addition, ponesimod prevented cuprizone-induced demyelination in the cingulum of an EAE mouse model, supporting protective and likely selective effects against demyelination in the brain (93).

In a phase 3, randomized, active comparator-controlled clinical trial, ARR, number of MRI brain lesions, and brain volume loss at week 108 was lower with ponesimod compared with teriflunomide in participants with RMS (34). Cognitive processing speed and auditory processing speed were assessed with the SDMT and PASAT-3, respectively. Mean changes in SDMT and PASAT-3 scores from baseline to week 108 were numerically higher at most visits through week 108 in ponesimod-treated participants versus those treated with teriflunomide (94). Although ponesimod treatment did not impact time to 12- or 24-week confirmed disability accumulation compared with teriflunomide in participants with RMS, change from baseline to week 108 in MSFC z-scores favored ponesimod (34, 94). To our knowledge, studies utilizing MTR/MWF to assess the effects of ponesimod on remyelination are not published.

#### Siponimod

Siponimod is indicated for the treatment of adults with RMS, including CIS, RRMS, and active SPMS in the US and for adults with active SPMS in the EU (95). Siponimod is an S1P receptor modulator that binds with high affinity to S1P_1_ and S1P_5_ and blocks the capacity of lymphocytes to egress from lymph nodes, reducing the number of lymphocytes in the peripheral blood (95).

Siponimod is lipophilic and crosses the BBB, reaching dose-proportional steady-state levels in blood, concomitant with 6- to 8-fold higher levels in mouse brain homogenates after 10 days of treatment (96, 97). In preclinical studies, siponimod decreased oligodendrocyte cell death and axon demyelination, stimulated remyelination, prevented neurons from astrocyte-induced degeneration, attenuated astrogliosis and microgliosis, and induced proregenerative microglia (96, 98–101). In addition, siponimod provided neuroprotective benefits in murine EAE, independent of peripheral immune effects, suggesting direct CNS effects (96, 102).

In a phase 3, randomized clinical trial in SPMS, siponimod slowed disability progression, decreased ARR, reduced the accumulation of MRI brain lesions, and reduced the rate of brain atrophy compared with placebo (35). Of the five participants with SPMS treated with siponimod from the phase 3 study who consented to CSF sampling, all had siponimod (low nM range) in their CSF (97). *Post hoc* analyses demonstrated positive effects on cognition, as siponimod improved SDMT scores from baseline at months 12 and 24 compared with placebo (103). Most notably, siponimod had a significant and consistent impact on MTR decrease in normal-appearing white matter and cortical grey matter over time compared with placebo, while improving MTR recovery in newly formed lesions, consistent with possible remyelination (104). In addition, siponimod treatment improved brain tissue integrity/myelination within newly formed normalized MTR lesions across brain tissues compared with placebo (105).

### Direct impact of DMTs on the CNS

Pivotal active-comparator phase 3 clinical trials established greater efficacy in measures of brain volume, cognition, and/or disability for DMTs acting directly on the CNS (e.g., fingolimod, ozanimod, and ponesimod) than those that may be limited to the periphery (e.g., teriflunomide and IFN β-1a) in participants with MS (31–34). Fingolimod treatment, however, did not slow disease progression compared with placebo in a phase 3 clinical trial of participants with primary progressive MS, suggesting that this anti-inflammatory therapy for relapse-onset MS is less likely to be effective in primary progressive disease, and other treatment approaches may be needed for these patients (106). In addition, other putative remyelination-promoting therapies, including opicinumab, MD-1003, and elezanumab failed to meet their study endpoints in MS clinical trials; lack of efficacy may have been due to the studied dose, time of treatment initiation, proof of concept study endpoints, or patient selection (107–110). The impact of centrally acting DMTs on brain volume, cognition, and/or disability contrasts with the lack of observed efficacy of remyelinating therapies and warrants further investigation.

### Future perspectives

The MS treatment landscape is continuously evolving, and ublituximab (an anti-CD20 monoclonal antibody) was recently approved by the FDA (111). Other therapies that target novel pathways are also in development (e.g., autologous hematopoietic stem cell transplantation [aHSCT], intrathecal stem cells, Bruton’s tyrosine kinase [BTK] inhibitors, clemastine fumarate, Epstein Barr virus [EBV]-targeted T cell immunotherapy, and temelimab) (112–122); preliminary data for these novel therapies are suggestive of CNS effects, as summarized in more detail below.

An observational, multicenter study provided Class IV evidence for aHSCT as an immunosuppressant MS therapy, because treatment induced durable disease remission for over 10 years in most participants with RRMS (including extremely aggressive forms of MS) (121). A randomized, phase 3 clinical trial (NCT04047628) is underway to determine the efficacy of aHSCT in participants with RMS, RRMS, or SPMS (123). Similarly, a pilot study of intrathecal mesenchymal stem cells in participants with RRMS or SPMS found that treatment stalled the disease progression of SPMS, and a phase 1/2 study (NCT04749667) is recruiting to determine efficacy in participants with progressive MS (112, 124).

BTK inhibitors primarily prevent B cell activation and maturation and are considered a promising therapeutic treatment option for MS that could target CNS-compartmentalized B cells, macrophages, and microglia (125). In a murine EAE model, the BTK inhibitors evobrutinib and tolebrutinib crossed the BBB (126, 127), with other preclinical studies showing BTK inhibitors to target CNS-inflammatory processes, including the reduction of microglial activation and inflammatory signaling (128–130). In addition, BTK inhibition has a positive effect on remyelination by targeting microglia, as demonstrated in *ex vivo* and *in vivo* models (131). A phase 1 trial found that evobrutinib was well tolerated in healthy volunteers, and a phase 2 trial found that treatment reduced the total number of GdE lesions compared with placebo but did not have an effect on relapse rate or disability progression in participants with RMS (118, 122). In a phase 1 tolebrutinib clinical trial, the CSF:plasma ratio of free tolebrutinib was 2.25 and treatment was considered well tolerated in healthy volunteers (116). A phase 2b trial in participants with RMS found that tolebrutinib treatment reduced the total number of GdE lesions compared with placebo (51). Additional phase 2 and phase 3 clinical trials are planned/underway for evobrutinib (NCT02975349, NCT04338022, and NCT04338061) and tolebrutinib (NCT04411641, NCT04410991, NCT04410978, and NCT04458051), as well as other BTK inhibitors, including fenebrutinib (NCT05119569, NCT04586010, NCT04586023, and NCT04544449), orelabrutinib (NCT04711148), remibrutinib (NCT05147220 and NCT05156281), and BIIB061 (NCT04079088) for relapsing and progressive forms of MS (118, 132–145).

Clemastine fumarate, originally developed as a first-generation antihistamine, is a muscarinic receptor antagonist that crosses the BBB and may promote remyelination (146). In the randomized, placebo-controlled crossover ReBUILD study, clemastine fumarate reduced visual-evoked potential latency delay in participants with RMS and chronic demyelinating optic neuropathy (120) and is considered the first randomized, controlled MS trial to demonstrate efficacy of a remyelinating agent (120). Phase 1 and 2 trials are planned/underway to assess the effects of clemastine fumarate as a monotherapy or in combination with metformin on remyelination in participants with relapsing or progressive forms of MS (147, 148).

EBV-targeted T cell immunotherapies have the potential to target cells associated with MS pathophysiology and may have a role in the CNS, as T cells access all compartments within the CNS (114). A case report of a patient with secondary progressive MS treated with an EBV-targeted T cell immunotherapy showed clinical improvement with reduced disease activity via MRI and decreased intrathecal immunoglobulin production (115). An open-label phase 1 trial of 10 participants with primary progressive multiple sclerosis and SPMS found EBV-targeted T cell treatment to be associated with clinical improvement in 7 participants, with 3 having improved EDSS score (114). Retrospective analysis of participants with progressive MS in part 1 of EMBOLD (NCT03283826), a single-arm phase 1 study with an open-label extension, found long-term disability improvement during ATA188 treatment (investigational EBV-targeted T cell immunotherapy) to be related to brain volume change and MTR in T2 lesions, suggesting the potential for remyelination over time (117). Part 2 of EMBOLD is a double-blind, randomized, placebo-controlled trial that is currently underway (149).

Early data for the immunoglobulin G4 monoclonal antibody temelimab suggest CNS effects on MRI measures of neurodegeneration (119). Although a randomized, placebo-controlled phase 2b study of temelimab failed to show an effect on acute inflammation, radiologic signs of possible neural protective effects suggest the potential use of temelimab in progressive MS (119).

In future clinical trials, applying appropriate endpoints that distinguish between peripheral and direct CNS effects may guide the therapeutic landscape for MS. A molecule is commonly considered to penetrate the BBB if the brain:plasma ratio is >0.04 using nonperfused brain tissue; however, this single parameter does not guarantee that adequate brain penetration is achieved for ligand-target interaction for a specific compound and should be accompanied by assessment of therapeutic response (150). Assessing the therapeutic response in the CNS may be accomplished with biomarkers (e.g., neurofilament light chain and glial fibrillary acidic protein), monitoring SELs and/or paramagnetic rim lesions, myelin measurements (e.g., MTR/MWF and ultrashort echo time MRI), P100 latency using multifocal or full-field visual evoked potential, optical coherence tomography, or *in vivo* imaging techniques (e.g., positron emission tomography imaging of microglia). The reliability of these techniques for determining direct effects within the CNS remains under investigation (110, 151–157). Well-designed clinical trials that consider these approaches are needed to substantiate new treatment modalities for MS that may include combining BBB-penetrating DMTs with peripherally acting monoclonal antibodies to limit disease progression and improve treatment outcomes through promoting immunomodulation, remyelination, and neuroprotection (158, 159).

## Conclusions

Of the DMTs currently available for the treatment of MS, data for cladribine, fingolimod, ozanimod, ponesimod, and siponimod are suggestive of direct CNS effects. Future MS studies may show a broader therapeutic benefit for DMTs that directly impact the CNS than those that act on the peripheral immune response alone.

## Author contributions

H-PH: Writing – review & editing, Conceptualization. BACC: Writing – review & editing, Conceptualization. MB: Writing – review & editing, Conceptualization. SGM: Writing – review & editing, Conceptualization. AB-O: Writing – review & editing, Conceptualization. LS: Writing – review & editing, Conceptualization.
