# Supplementary material for: Bioavailable central nervous system disease-modifying therapies for multiple sclerosis
Source: Front Immunol. 2023 Nov 29;14:1290666. doi: 10.3389/fimmu.2023.1290666 (PMC10755740; doi:10.3389/fimmu.2023.1290666)
Supplement: Supplementary file 1 [file Table_1.docx]

**Supplemental Material**

**Bioavailable Central Nervous System Disease-Modifying
Therapies for Multiple Sclerosis**

Hans-Peter Hartung, MD; Bruce A.C. Cree, MD, PhD, MAS; Michael Barnett, MBBS, PhD; Sven G. Meuth, MD, PhD;
Amit Bar-Or, MD, BSC; Lawrence Steinman, MD

| **Table 1 DMTS With Insufficient Evidence of Direct CNS Effects in MS** | | | | | | |
| --- | --- | --- | --- | --- | --- | --- |
|  | **Preclinical evidence** | **Ability to traverse the BBB** | **Phase 3 clinical evidence^a^** | | | **MTR/MWF assessments** |
|  |  |  | **Brain volume** | **Cognition** | **Disability** |  |
| **Anti-CD52 monoclonal antibody** | | | | | | |
| **Alemtuzumab** | Elicited differential effects on the phenotype and function of microglia in the CNS of EAE mice (1)  Protected neurons and potentially promoted remyelination in EAE mice (2)  Depleted B-cell aggregates in the CNS of EAE mice (3) | Unable to cross the BBB (4) | Reduced median % change in BPF volume loss over 24 months compared with IFN β-1a in participants with RRMS (5)  Treatment naive  12 mg: −0.87%, *P* < .0001 vs IFN β-1a  IFN β-1a: −1.49%  Treatment-experienced (inadequate response)  12 mg: −0.62%, *P* = .012 vs IFN β-1a  IFN β-1a: −0.81% | NR | No significant difference in rate of sustained accumulation of disability over 6 months or mean change in EDSS or MSFC scores from baseline to month 24 in treatment-naive participants with RRMS (6)  Reduced the risk of sustained disability accumulation confirmed over 6 months from baseline and improved mean (95% CI) change in EDSS scores from baseline to 24 months compared with IFN β-1a in participants with RRMS and an inadequate response to prior therapy; no significant difference in MSFC scores (7)  Risk reduction in sustained disability accumulation  12 mg: 42%, *P* = .0084 vs IFN β-1a  EDSS  12 mg: −0.17 (−0.29 to −0.05), *P* < .0001  IFN β-1a: 0.24 (0.07 to 0.41) | Stabilized MWF in NAWM and T2 lesions over 24 months in participants with RRMS (8)  Stabilized MTR mean changes in NAGM, WM, and lesional MTR over 3 years in participants with early RRMS (9) |
| ***Nrf2 activators*** | | | | | | |
| **Dimethyl, diroximel, and monomethyl fumarates^b^** | Inhibited microglial and astroglial activation and subsequent release of proinflammatory mediators in an in vitro model of brain inflammation (10)  Protected oligodendrocytes from stress-induced overproduction of reactive oxygen species, apoptosis, and autophagy in a murine model (11) | T_max_ of monomethyl fumarate in CSF peaked 2 hours after that of plasma, with 11% measured in the CSF in participants with SPMS (12) | Prevented substantial adjusted annual mean percentage changes in BV over 6 years in participants with RRMS (13)  DMF (phase 3) to DMF (extension): −1.32%; range, −1.60% to −1.05%  PBO (phase 3) to DMF (extension): −1.36%; range, −1.68% to −1.04% | NR | No significant differences were observed in reduction of risk for 12- or 24-week CDP over the 24-month study between DMF and PBO in participants with RRMS (14)  Reduced the risk of 12-week CDP over 24 months compared with PBO in participants with RRMS (15)  Participants with sustained 12-week CDP:  240 mg BID: 38%; HR, 0.62, 95% CI, 0.44 to 0.87, *P* = .005 vs PBO  240 mg TID: 34%; HR, 0.66, 95% CI, 0.48 to 0.92, *P* = .01 vs PBO | Increased percentage change in median whole brain and NABT MTR from baseline to 24 months compared with PBO in participants with RRMS (16)  No significant differences in MTR from baseline to month 24 compared with PBO in participants with RRMS (17) |
| ***Interferons*** | | | | | | |
| **IFN β^c^** | NR | Unable to cross the BBB^d^ (18) | (IFN β-1a) Resulted in less mean percentage changes in BPF during year 2 compared with PBO in participants with RRMS (19)  6 MIU QWK: −0.233, *P* = .03 vs PBO  PBO: −0.521  (peg IFN β-1a) No significant difference in WBV compared with PBO in participants with RRMS (20)  (peg IFN β-1a) Reduced mean % change in WBV from week 24 to week 96 compared with a delayed treatment group in participants with RRMS (21) | (IFN β-1b) Increased mean ± SD PASAT-3 scores at year 2 in participants with CIS compared with PBO (22)  250 µg QOD: 2.3 ± 6.3, *P* = .018 vs PBO  PBO: 0.8 ± 5.5 | (IFN β) No significant difference in clinical disability status scores over 1.5 years compared with PBO in participants with MS (23)^e^  (IFN β-1a) Increased time to disability progression from baseline to week 104 compared with PBO in participants with RMS (*P* = .02) (24)  (IFN β-1a) Resulted in lower confirmed and sustained EDSS scores from baseline to week 104 compared with PBO in participants with RMS (*P* = .02) (24)  (peg IFN β-1a) Reduced the proportion of 12-week sustained disability progression at 48 weeks compared with PBO in participants with RRMS (20)  125 µg q2wk: 0.068; HR, 0.62, 95% CI, 0.4 to 0.97, *P* = .0383 vs PBO  125 µg q4wk: 0.068; HR, 0.62, 95% CI, 0.4 to 0.97, *P* = .0380 vs PBO  PBO: 0.105  (IFN β-1b) No significant difference in EDSS scores at 36 months compared with PBO in participants with RRMS (25)^e^  (IFN β-1b) Increased the probability of remaining free from disability progression over 33 months compared with PBO in participants with SPMS (*P* = .0008) (26)  (IFN β-1b) Reduced the proportion of participants with confirmed EDSS progression compared with PBO in participants with SPMS (26)  8 MIU QOD: 38.9%, *P* = .0048 vs PBO  PBO: 49.7%  (IFN β-1b) No significant difference in time to confirmed progression of EDSS scores at year 3 compared with PBO in participants with SPMS (27)^e^ | (IFN β-1a) Influenced the rate of recovery of low MTR following the appearance of new GdE lesions in participants with RRMS (28)  (peg IFN β-1a) Lowered mean % decrease in MTR compared with PBO in participants with RRMS (125 µg q2wk dose only) (20) |
| ***T-cell suppressor*** | | | | | | |
| **Glatiramer acetate** | Increased the proliferation, differentiation, and survival of OPCs in an EAE mouse model (29)  Promoted oligodendrogenesis and indices of remyelination in mice subjected to lysolecithin-induced demyelination (30) | Unable to cross the BBB (31) | No significant difference in % change in BV from baseline to the last observed value of the study in BVL compared with PBO in participants with CIS (32)  No significant difference in % change of BV from baseline to month 12 compared with PBO in participants with RRMS (33) | No significant difference in 10/36 spatial recall, PASAT, SDMT, word list generation, or Buschke selective reminding test from baseline to month 24 compared with PBO in participants with RRMS (34) | Improved mean ± SD EDSS score change from baseline to month 24 compared with PBO in participants with RRMS (35)  20 mg: −0.05 ± 1.13, *P* = .023 vs PBO  PBO: 0.21 ± 0.99  No significant difference in 3-month CDP after 24 months in participants with RRMS compared with PBO (35) | Increased average postlesion FSMTR recovery in GdE and ΔFSMTR lesions compared with IFN β-1b in participants with MS (36) |
| ***DNA-damage repair inhibitor*** | | | | | | |
| **Mitoxantrone** | Repressed the production of astrocyte-induced cytotoxic molecules in vitro (37)  Enhanced remyelination and protected from demyelination in an EAE rat model (38)  Repressed markers of microglial activation and inflammation in vitro (39) | Does not cross the BBB (40) | NR | NR | Decreased mean ± SD EDSS scores from baseline to month 24 compared with PBO in participants with SPMS (41)  12 mg/m^2^: −0.13 ± 0.90; difference from PBO (95% CI), 0.24 (0.04 to 0.44); *P* = .0194 vs PBO  PBO: 0.23 ± 1.01  Reduced the proportion of participants with CDP over 2 years compared with PBO in participants with RRMS (42)^e^  8 mg/m^2^: 7%; difference vs PBO, 30, 95% CI, 8 to 52  PBO: 38% | NR |
| ***α4-integrin receptor antagonist*** | | | | | | |
| **Natalizumab** | NR | Unable to cross the BBB (4) | No significant difference in mean % change in WBV from week 24 to week 96 compared with PBO in participants with SPMS (43)  Reduced mean % reduction in BPF over 2 years compared with PBO in participants with RMS (44)  300 mg: 0.56%, *P* = .002 vs PBO  PBO: 0.40% | Reduced the risk of confirmed progression of cognitive deficits (0.5 SD change in PASAT-3 scores) over 2 years compared with PBO in participants with RMS (45)^e^  300 mg: −43%; HR, 0.57, 95% CI, 0.37 to 0.89, *P* = .013 vs PBO | Reduced the risk of sustained disability progression by 42% over 2 years in participants with RMS compared with PBO (46)  300 mg: HR, 0.58, 95% CI, 0.43 to 0.77, *P* < .001 vs PBO | Increased the volume of tissue with increased white matter MTR at 2 years compared with IFN-β 1a in participants with RRMS/RSPMS (*P* = .008) (47) |
| ***Anti-CD20 monoclonal antibody*** | | | | | | |
| **Ocrelizumab** | NR | Unable to cross the BBB (4) | Nonconfirmatory or nonsignificant differences in the mean % of BV from week 24 to week 96 compared with IFN β-1a in participants with RMS (48)  Lowered the adjusted mean % change in BV from week 24 to week 120 compared with PBO in participants with PPMS (49)  600 mg: −0.90%, *P* = .02 vs PBO  PBO: −1.09% | Improved mean ± SE SDMT scores from baseline over 96 weeks compared with IFN β-1a in participants with RMS (50)  600 mg: 6.2 ± 1.2, *P* = .023 vs IFN β-1a  IFN β-1a: 2.6 ± 1.2 | Reduced the proportion of participants with 12-week CDP over 96 weeks in participants with RMS compared with IFN β-1a(48)  600 mg: 9.1%; HR, 0.6, 95% CI, 0.45 to 0.81, relative risk reduction, 40%, *P* < .001 vs IFN β-1a  IFN β-1a: 13.6%  Lowered the proportion of participants with 24-week CDP over 96 weeks in participants with RMS compared with IFN β-1a (48)  600 mg: 6.9%; HR, 0.6, 95% CI 0.43 to 0.84, relative risk reduction, 40%, *P* = .003 vs IFN β-1a  IFN β-1a: 10.5%  Increased the % of participants with 12-week CDI over 96 weeks in participants with RMS compared with IFN β-1a (48)  600 mg: 20.7%, *P* = .02 vs IFN β-1a  IFN β-1a: 15.6%  Reduced the % of participants with 12-week CDP over 216 weeks compared with PBO in participants with PPMS (49)  600 mg: 32.9%; HR, 0.76, 95% CI, 0.59 to 0.98, relative risk reduction, 24%, *P* = .03 vs PBO  PBO: 39.3%  Reduced the % of participants with 24-week CDP over 216 weeks compared with PBO in participants with PPMS (49)  600 mg: 29.6%; HR, 0.75, 95% CI, 0.58 to 0.98, relative risk reduction, 25%, *P* = .04 vs PBO  PBO: 35.7% | Prevented demyelination in NAWM and in chronic lesions compared with IFN β-1a in participants with RMS (51) |
| **Ofatumumab** | NR | Unable to cross the BBB (4) | No significant difference in annual rate of BVL from baseline up to 30 months compared with teriflunomide in participants with RMS (52) | Increased mean ± SE SDMT score changes from baseline to month 24 compared with teriflunomide in participants with RMS (53)  20 mg: 3.50 ± 0.358, *P* = .030 vs teriflunomide  Teriflunomide: 2.39 ± 0.365 | Reduced the proportion of participants with 3-month CDP over 24 months compared with teriflunomide in participants with RMS (52)  20 mg: 10.9%; HR, 0.66, 95% CI, 0.50 to 0.86, *P* = .002 vs teriflunomide  Teriflunomide: 15%  Reduced the proportion of participants with 6-month CDP over 24 months compared with teriflunomide in participants with RMS (52)  20 mg: 8.1%; HR, 0.68, 95% CI, 0.50 to 0.92, *P* = .01 vs teriflunomide  Teriflunomide: 12%  No significant difference in CDI over 24 months compared with teriflunomide in participants with RMS(52) | NR |
| ***Dihydroorotate dehydrogenase inhibitor*** | | | | | | |
| **Teriflunomide** | Promoted oligodendroglial cell differentiation in vitro (54)  Reduced astrocytic inflammatory responses in vitro (55)  Increased OPC proliferation and differentiation in vitro and promoted remyelination in vivo (56) | Low permeability; 1%–2% of the serum concentration reaches the CNS (57) | Reduced median % change in BVL from baseline to years 1 and 2 compared with PBO in participants with RMS (58)  14 mg  Year 1: 0.39%; relative reduction, 36.9%, *P* = .0001 vs PBO  Year 2: 0.90%; relative reduction, 30.6, *P* = .0001 vs PBO  PBO  Year 1: 0.61%  Year 2: 1.29% | Improved LS mean PASAT-3 scores from baseline to week 96 compared with PBO in participants with RMS (59)  14 mg: 0.09; difference, 0.11 (0.05), *P* = .0146 vs PBO  PBO: −0.03 | Reduced the risk of sustained disability accumulation over 132 weeks compared with PBO in participants with RMS (60)  14 mg: Risk reduction, 31.5%; 95% CI, −0.4 to 53.3; log-rank *P* = .0442 vs PBO  Increased the proportion of participants free from disability accumulation after 48 weeks compared with PBO in participants with RMS (60)  14 mg: 92.2%, 95% CI, 89.2 to 95.1  PBO: 85.8%; 95% CI, 82.1 to 89.4  Reduced LS mean ± SE EDSS scores from baseline to week 48 compared with PBO in participants with RMS (60)  14 mg: −0.05 ± 0.05, *P* = .0429 vs PBO  PBO: 0.09 ± 0.05  Reduced the risk of sustained disability accumulation over 108 weeks compared with PBO in participants with RMS (61)  14 mg: risk reduction, 29.8%, *P* = .03 vs PBO | NR |

^a^Includes properly powered, randomized phase 3 clinical trials, corresponding to a quality of evidence of 1 per the Oxford Centre for Evidence-Based Medicine, as well as post hoc analyses and open-label extensions of phase 3 trials.

^b^Efficacy data for monomethyl fumarate are based on bioequivalence with, preapproval clinical trial data for, and postmarket monitoring of patients treated with dimethyl fumarate (62); diroximel fumarate results in monomethyl fumarate systemic exposure that is bioequivalent to dimethyl fumarate, thus, diroximel fumarate and dimethyl fumarate have similar efficacy and safety profiles (63-65).

^c^Data from BENEFIT (participants with a first event suggestive of multiple sclerosis) were included only if other clinical trials in participants with RMS or PMS were not reported.

^d^On the basis of IFN alpha data (18).

^e^Phase unspecified.

Abbreviations: BBB, blood-brain barrier; BID, 2 times a day; BPF, brain parenchymal fraction; BV, brain volume; BVL, brain volume loss; CDI, confirmed disability progression; CDP, continued disability progression; CI, confidence interval; CIS, clinically isolated syndrome; CNS, central nervous system; CSF, cerebrospinal fluid; DMF, dimethyl fumarate; EAE, experimental autoimmune encephalomyelitis; EDSS, Expanded Disability Status Scale; FSMTR, fat saturation producing a magnetization transfer effect used to creative a fat saturation ratio image; GdE, gadolinium-enhancing; HR, hazard ratio; IFN, interferon; LS, least-squares; MS, multiple sclerosis; MSFC, Multiple Sclerosis Functional Composite; MTR, magnetization transfer ratio; MWF, myelin water fraction; NABT, normal appearing brain tissue; NAGM, normal appearing- grey matter; NAWM, normal-appearing white matter; NR, not reported in the literature to our knowledge; Nrf2, nuclear factor erythroid 2–related factor 2; OPC, oligodendrocyte precursor cell*,* PASAT, Paced Auditory Serial Addition Test; PBO, placebo; PMS, progressive multiple sclerosis; PPMS, primary progressive multiple sclerosis; QOD, every other day; QWK, every week; RMS, relapsing multiple sclerosis; RRMS, relapsing-remitting multiple sclerosis; RSPMS, relapsing secondary progressive multiple sclerosis; SD, standard deviation; SDMT, Symbol Digit Modalities Test; SE, standard error; SPMS, secondary progressive multiple sclerosis; TID, 3 times a day; T_max_, time to peak drug concentration; WM, white matter; WBV, whole brain volume.

| **Table 2 DMTs With Evidence of Direct CNS Effects in MS** | | | | | | |
| --- | --- | --- | --- | --- | --- | --- |
|  | **Preclinical evidence** | **Ability to traverse the BBB** | **Phase 3 clinical evidence^a^** | | | **MTR/MWF assessments** |
|  |  |  | **Brain volume** | **Cognition** | **Disability** |  |
| ***DNA synthesis inhibitor*** | | | | | | |
| **Cladribine** | Reduced the granularity, phagocytotic ability, and motility of LPS-stimulated microglia in vitro using concentrations overlapping with CSF concentrations in humans (66)  Induced apoptosis and inhibited the proliferation of microglial cells (67), thereby reducing the production of proinflammatory cytokines in vitro (67, 68) | Mean ± SD concentration of 6.1 ± 4.0 and a CSF:plasma concentration ratio of 18.2% ± 10 (69) | Reduced mean ± SD annualized % BV change per year (months 6–24) over 2 years compared with PBO in participants with RMS (70)  3.5 mg/kg: −0.56% ± 0.68, *P*= .010 vs PBO  5.25 mg/kg: −0.57% ± 0.72, *P* = .019 vs PBO  PBO: −0.70% ± 0.79% | NR | Lowered the risk of 3-month sustained disability progression at 96 weeks compared with PBO in participants with RMS (71)  3.5 mg: HR, 0.67, 95% CI, 0.48 to 0.93, *P* = .02 vs PBO  5.25 mg: HR, 0.69, 95% CI, 0.49 to 0.96, *P* = .03 vs PBO | NR |
| ***S1P receptor modulators*** | | | | | | |
| **Fingolimod** | Reduced S1P_1_ signaling from astrocytes in an EAE mouse model (72)  Promoted the proliferation and differentiation of OPCs (73)  Ameliorated pathological effectors associated with microglial activation, leading to an increase in protein and morphological markers of remyelination following lysophosphatidylcholine treatment in vitro (74)  Enhanced the expression of BDNF through direct effects on neurons in vitro (75) | Radiolabeled fingolimod crossed the BBB and accumulated in the white matter in the CNS of an EAE rat model (76)  In humans, following intravenous injection, a radiolabeled fingolimod analog entered the brain, with uptake steadily increasing up to 26 h after dosing (77) | Reduced mean ± SD rate of BVL from baseline compared with PBO at 12 and 24 months or IFN β-1a at 12 months in participants with RMS (78, 79)  12 months  1.25 mg: −0.44 ± 1.08, *P*< .001 vs PBO  0.5 mg: −0.50 ± 1.05, *P*< .03 vs PBO  PBO: −0.65 ± 1.50  24 months  1.25 mg: −0.89 ± 1.39, *P*< .001 vs PBO  0.5 mg: −0.84 ± 1.31, *P*< .001 vs PBO  PBO: −1.31 ± 1.50  12 months  1.25 mg: −0.30 ± 0.65, *P*< .001 vs IFN β-1a  0.5 mg: −0.31 ± 0.65, *P*< .001 vs IFN β-1a  IFN β-1a: −0.45 ± 0.73  Reduced mean ± SD % BV change from baseline to month 12 compared with PBO in participants with RRMS (80)  1.25 mg: −0.35% ± 1.20, *P*< .0001 vs PBO  0.5 mg: −0.38% ± 0.97, *P*= .0004 vs PBO  PBO: −0.63% ± 1.05 | Improved adjusted mean PASAT-3 scores from baseline at 6, 12, and 24 months, with a sustained effect up to 120 months, in participants with RRMS compared with PBO (81)  6-month  between-group difference: 1.3, *P*= .0007 vs PBO  12-month  between-group difference: 1.1, *P*= .0044 vs PBO  24-month between‑group difference: 1.1, *P*= .0028 vs PBO | Improved/stabilized mean ± SD EDSS and MSFC z scores over 24 months compared with PBO in participants with RMS (78)  EDSS  1.25 mg:−0.03 ± 0.88, *P* = .002 vs PBO  0.5 mg: 0.00 ± 0.88, *P*= .002 vs PBO  PBO: 0.13 ± 0.94  MSFC z scores  1.25 mg: 0.01 ± 0.40, *P* = .02 vs PBO  .5 mg: 0.03 ± 0.39, *P*= .01 vs PBO  PBO: −0.06 ± 0.57  Reduced the risk of 3-month and 6-month disability progression in the 24-month study in participants with RMS compared with PBO (78)  3 months  1.25 mg: HR, 0.68; 95% CI, 0.50 to 0.93*, P* = .02 vs PBO  0.5 mg: HR, 0.70; 95% CI, 0.52 to 0.96*, P* = .02 vs PBO  6 months 1.25 mg: HR, 0.60; 95% CI, 0.41 to 0.86*, P*= .006 vs PBO  0.5 mg: HR, 0.63; 95% CI, 0.44-0.90*, P*= .01 vs PBO  No significant differences in time to disability progression or in the proportion of participants with CDP over 12 months compared with IFN β-1a in participants with RMS (79)  No significant difference between fingolimod 0.5 or 1.25 mg on time to 3- or 6-month CDP in the 24-month study compared with PBO in participants with RRMS (80) | Enhanced tissue damage recovery in lesions as soon as 6 months and after 2 years in NAWM and GM in participants with RRMS (82) |
| **Ozanimod** | Reduced clinical scores in an EAE mouse model at a dose that did not induce lymphopenia (83)  Reduced axonal breaks and improved functional capabilities following cuprizone-induced demyelination in an EAE mouse model (84)  Ex vivo treatment of EAE corticostriatal slices with ozanimod increased the mRNA expression of a marker of microglia activation and decreased the expression of other inflammatory markers (85)  Elicited potent AKT and ERK phosphorylation in human astrocytes in vitro (86) | Brain:blood ratio of 10:1 and 16:1 in mice and rats, respectively (83) | Slowed mean ± SD WBV, CGMV, and TV loss from baseline to month 24 compared with IFN β-1a in participants with RMS (87)  WBV 0.46 mg: −0.71 ± 0.75, nominal *P* = .0002 vs IFN β-1a  0.92 mg: −0.71 ± 0.88, nominal *P* < .0001 vs IFN β-1a  IFN β-1a: −0.94 ± 0.94  CGMV 0.46 mg: −0.50 ± 0.80, nominal *P* < .0001 vs IFN β-1a  0.92 mg: −0.44 ± 0.86, nominal *P* < .0001 vs IFN β-1a  IFN β-1a: −1.11 ± 0.96  TV 0.46 mg: −1.50 ± 1.79, nominal *P* = .0133 vs IFN β-1a  0.92 mg: −1.4 ± 2.06, nominal *P* = .0004 vs IFN β-1a  IFN β-1a: −1.85 ± 1.97  Slowed mean ± SD WBV, CGMV, and TV loss from baseline to month 12 compared with IFN β-1a in participants with RMS (88)  WBV 0.46 mg: −0.49 ± 0.61, nominal *P* = .0092 vs IFN β-1a  0.92 mg: −0.41 ± 0.64, nominal *P* < .0001 vs IFN β-1a  IFN β-1a: −0.61 ± 0.69  CGMV 0.46 mg: −0.34 ± 0.79, nominal *P* < .0001 vs IFN β-1a  0.92 mg: −0.16 ± 0.872, nominal *P* < .0001 vs IFN β-1a  IFN β-1a: −1.00 ± 0.97  TV 0.46 mg: −1.12 ± 1.62, nominal *P* < .0001 vs IFN β-1a  0.92 mg: −1.12 ± 1.63, nominal *P* < .0001 vs IFN β-1a  IFN β-1a: −1.72 ±1.94 | Improved mean change ± SD SDMT z scores from baseline to month 12 compared with IFN β‑1a in participants with RMS (88)  0.46 mg: 0.06 ± 0.55, nominal *P* = .0246 vs IFN β-1a  0.92 mg: 0.07 ± 0.65, nominal *P* = .0024 vs IFN β-1a  IFN β-1a: −0.03 ± 0.51 | Improved mean change ± SD MSFC scores from baseline to month 24 compared with IFN β-1a in participants with RMS (87)  0.46 mg: 0.03 ± 0.48, nominal *P* = .0246 vs IFN β-1a  0.92 mg: −0.006 ± 0.78, NS vs IFN β-1a  IFN β-1a: −0.07 ± 0.75  No significant difference in the proportion of participants with 3- or 6-month CDP compared with IFN β-1a in 12–24-month studies in participants with RMS (87)  Sustained 3-month and 6-month CDP for up to 5-years in participants with RMS previously treated with ozanimod (0.46 mg or 0.92 mg) or IFN β-1a in phase 3 trials (89)  3 months  0.92 mg: 346/2494 (13.9%) had CDP  6 months  0.92 mg: 285/2494 (11.4%) had CDP | NR |
| **Ponesimod** | Dampened glial activation and subsequently CNS neuroinflammation in an EAE mouse model (90)  Prevented cuprizone- induced demyelination in the cingulum of an EAE mouse model; inhibited intracellular signals and neuroinflammatory response of astrocytes in vitro (91) | Crosses the BBB^b^ | Reduced LS mean % change in BVL loss at week 108 compared with teriflunomide in participants with RMS (92)  20 mg: −0.91% (−1.03 to −0.79); difference, 0.34 % points, 95% CL, 0.17 to 0.50, exploratory *P* < .001 vs teriflunomide  Teriflunomide:  −1.25% (−1.36 to −1.13) | Increased LS mean change in SDMT scores from baseline to week 108 compared with teriflunomide in participants with RMS (93)  20 mg: difference, 0.07, 95% CL, −1.00 to 1.14 vs teriflunomide  Increased mean numerical changes in PASAT-3 scores from baseline to week 108 at most visits compared with teriflunomide in participants with RMS (93) | No significant difference in risk of 12-week or 24-week CDA compared with teriflunomide in participants with SPMS (92)  Improved MSFC z-scores from baseline to week 108 compared with teriflunomide in participants with RMS (93)  20 mg: 0.02; mean difference, 0.059, *P* = .047 vs teriflunomide  Teriflunomide: −0.039 | NR |
| **Siponimod** | Decreased oligodendrocyte cell death and axon demyelination in an EAE mouse model (94)  Stimulated remyelination in Xenopus (95)  Prevented neurons from astrocyte-induced degeneration in vitro (96)  Attenuated astrogliosis and microgliosis and provided neuroprotective effects independent of peripheral immune effects in EAE mice (97, 98)  Increased proremyelination potential in a *Xenopus* model (99) | In mice, a siponimod-loaded diet over 10 days achieved dose-proportional steady-state levels of siponimod in blood, concomitant with 6- to 8-fold higher levels in brain homogenates (100)  In a randomized, PBO-controlled, phase 3 clinical study, all participants with SPMS who consented to CSF sampling had a low nM range of siponimod in their CSF (n = 5) (100) | Slowed adjusted mean % (95% CI) BVL over 12 and 24 months compared with PBO in participants with SPMS (101)  2 mg: −0.50% (−0.55 to −0.44), *P*= .0002 vs PBO  PBO  −0.65% (−0.72 to −0.58) | Improved SDMT scores from baseline at months 12 and 24 compared with PBO in participants with SPMS (102)  Month 12 2 mg: difference, 1.09; 95% CI, 0.23 to 1.94, *P*= .0132 vs PBO  Month 24  2 mg: difference, 2.30; 95% CI, 1.11 to 3.50, *P*= .0002 vs PBO | Reduced the risk of 3- and 6-month CDP compared with PBO in participants with SPMS (101)  3-month  2 mg: HR, 0.79; 95% CI, 0.65 to 0.95*, P*= .013 vs PBO  6-month  2 mg: HR, 0.74; 95% CI, 0.60 to 0.92*, P*= .0058 vs PBO | Reduced median normalized MTR across brain tissues from baseline to month 24 compared with PBO in participants with SPMS (103)  Improved brain tissue integrity/myelination within newly formed normalized MTR lesions across brain tissues over 24 months compared with PBO in participants with SPMS (104) |
| ^a^Includes properly powered, randomized phase 3 clinical trials, corresponding to a quality of evidence of 1 per the Oxford Centre for Evidence-Based Medicine, as well as post-hoc analyses and open-label extensions of phase 3 trials.  ^b^Assumed based on class.  Abbreviations: AKT, protein kinase B; BBB, blood-brain barrier; BDNF, brain-derived neurotrophic factor; BV, brain volume; BVL, brain volume loss; CDA, confirmed disability accumulation; CDP, continued disability progression; CGMV, cortical grey matter volume; CI, confidence interval; CL, confidence limit; CNS, central nervous system; CSF,  cerebrospinal fluid; EAE, experimental autoimmune encephalomyelitis; EDSS, Expanded Disability Status Scale; ERK, extracellular-regulated kinase; GM, grey matter; HR, hazard ratio; IFN, interferon; LPS, lipopolysaccharide; LS, least-squares; mRNA, messenger RNA; MSFC, Multiple Sclerosis Functional Composite; MTR, magnetization transfer ratio; MWF, myelin water fraction; NAWM, normal-appearing white matter; NR, not reported in the literature to our knowledge; NS, not significant; OPC, oligodendrocyte precursor cell; PASAT, Paced Auditory Serial Addition Test; PBO, placebo; RMS, relapsing multiple sclerosis; RRMS, relapsing-remitting multiple sclerosis; S1P, sphingosine-1 phosphate; SD, standard deviation; SDMT, Symbol Digit Modalities Test; SPMS, secondary progressive multiple sclerosis; TV, thalamic volume; WBV, whole brain volume. | | | | | | |

**References**

1. Barbour M, Wood R, Harte T, Bushell TJ, Jiang HR. Anti-Cd52 Antibody Treatment in Murine Experimental Autoimmune Encephalomyelitis Induces Dynamic and Differential Modulation of Innate Immune Cells in Peripheral Immune and Central Nervous Systems. *Immunology* (2022) 165(3):312-27. Epub 2021/11/27. doi: 10.1111/imm.13437.

2. Ellwardt E, Vogelaar CF, Maldet C, Schmaul S, Bittner S, Luchtman D. Targeting Cd52 Does Not Affect Murine Neuron and Microglia Function. *Eur J Pharmacol* (2020) 871:172923. Epub 2020/01/22. doi: 10.1016/j.ejphar.2020.172923.

3. Simon M, Ipek R, Homola GA, Rovituso DM, Schampel A, Kleinschnitz C, et al. Anti-Cd52 Antibody Treatment Depletes B Cell Aggregates in the Central Nervous System in a Mouse Model of Multiple Sclerosis. *J Neuroinflammation* (2018) 15(1):225. Epub 2018/08/14. doi: 10.1186/s12974-018-1263-9.

4. Avasarala J. Monoclonal Antibodies, Blood-Brain Barrier and Disability in Multiple Sclerosis: Time for Combination Therapies. *GHS Proc* (2016) 1(2):89-91.

5. Arnold DL, Fisher E, Brinar VV, Cohen JA, Coles AJ, Giovannoni G, et al. Superior Mri Outcomes with Alemtuzumab Compared with Subcutaneous Interferon Β-1a in Ms. *Neurology* (2016) 87(14):1464-72. Epub 2016/09/04. doi: 10.1212/wnl.0000000000003169.

6. Cohen JA, Coles AJ, Arnold DL, Confavreux C, Fox EJ, Hartung HP, et al. Alemtuzumab Versus Interferon Beta 1a as First-Line Treatment for Patients with Relapsing-Remitting Multiple Sclerosis: A Randomised Controlled Phase 3 Trial. *Lancet* (2012) 380(9856):1819-28. doi: 10.1016/S0140-6736(12)61769-3.

7. Coles AJ, Arnold DL, Bass AD, Boster AL, Compston DAS, Fernández Ó, et al. Efficacy and Safety of Alemtuzumab over 6 Years: Final Results of the 4-Year Care-Ms Extension Trial. *Ther Adv Neurol Disord* (2021) 14. Epub 2021/05/27. doi: 10.1177/1756286420982134.

8. Vavasour IM, Tam R, Li DK, Laule C, Taylor C, Kolind SH, et al. A 24-Month Advanced Magnetic Resonance Imaging Study of Multiple Sclerosis Patients Treated with Alemtuzumab. *Mult Scler* (2019) 25(6):811-8. Epub 2018/04/18. doi: 10.1177/1352458518770085.

9. Button T, Altmann D, Tozer D, Dalton C, Hunter K, Compston A, et al. Magnetization Transfer Imaging in Multiple Sclerosis Treated with Alemtuzumab. *Mult Scler* (2013) 19(2):241-4. Epub 2012/04/12. doi: 10.1177/1352458512444915.

10. Wilms H, Sievers J, Rickert U, Rostami-Yazdi M, Mrowietz U, Lucius R. Dimethylfumarate Inhibits Microglial and Astrocytic Inflammation by Suppressing the Synthesis of Nitric Oxide, Il-1beta, Tnf-Alpha and Il-6 in an in-Vitro Model of Brain Inflammation. *J Neuroinflammation* (2010) 7:30. Epub 2010/05/21. doi: 10.1186/1742-2094-7-30.

11. Zarrouk A, Nury T, Karym EM, Vejux A, Sghaier R, Gondcaille C, et al. Attenuation of 7-Ketocholesterol-Induced Overproduction of Reactive Oxygen Species, Apoptosis, and Autophagy by Dimethyl Fumarate on 158n Murine Oligodendrocytes. *J Steroid Biochem Mol Biol* (2017) 169:29-38. Epub 2016/02/28. doi: 10.1016/j.jsbmb.2016.02.024.

12. Edwards KR, Kamath A, Button J, Kamath V, Mendoza JP, Zhu B, et al. A Pharmacokinetic and Biomarker Study of Delayed-Release Dimethyl Fumarate in Subjects with Secondary Progressive Multiple Sclerosis: Evaluation of Cerebrospinal Fluid Penetration and the Effects on Exploratory Biomarkers. *Mult Scler Relat Disord* (2021) 51:102861. Epub 2021/03/28. doi: 10.1016/j.msard.2021.102861.

13. Gold R, Arnold DL, Bar-Or A, Fox RJ, Kappos L, Chen C, et al. Safety and Efficacy of Delayed-Release Dimethyl Fumarate in Patients with Relapsing-Remitting Multiple Sclerosis: 9 Years' Follow-up of Define, Confirm, and Endorse. *Ther Adv Neurol Disord* (2020) 13. Epub 2020/05/20. doi: 10.1177/1756286420915005.

14. Fox RJ, Miller DH, Phillips JT, Hutchinson M, Havrdova E, Kita M, et al. Placebo-Controlled Phase 3 Study of Oral Bg-12 or Glatiramer in Multiple Sclerosis. *N Engl J Med* (2012) 367(12):1087-97. doi: 10.1056/NEJMoa1206328

15. Gold R, Kappos L, Arnold DL, Bar-Or A, Giovannoni G, Selmaj K, et al. Placebo-Controlled Phase 3 Study of Oral Bg-12 for Relapsing Multiple Sclerosis. *N Engl J Med* (2012) 367(12):1098-107. doi: 10.1056/NEJMoa1114287.

16. Arnold DL, Gold R, Kappos L, Bar-Or A, Giovannoni G, Selmaj K, et al. Magnetization Transfer Ratio in the Delayed-Release Dimethyl Fumarate Define Study. *J Neurol* (2014) 261(12):2429-37. Epub 2014/10/02. doi: 10.1007/s00415-014-7504-7.

17. Miller DH, Fox RJ, Phillips JT, Hutchinson M, Havrdova E, Kita M, et al. Effects of Delayed-Release Dimethyl Fumarate on Mri Measures in the Phase 3 Confirm Study. *Neurology* (2015) 84(11):1145-52. Epub 2015/02/15. doi: 10.1212/wnl.0000000000001360.

18. Smith RA, Norris F, Palmer D, Bernhardt L, Wills RJ. Distribution of Alpha Interferon in Serum and Cerebrospinal Fluid after Systemic Administration. *Clin Pharmacol Ther* (1985) 37(1):85-8. Epub 1985/01/01. doi: 10.1038/clpt.1985.16.

19. Rudick RA, Fisher E, Lee JC, Simon J, Jacobs L. Use of the Brain Parenchymal Fraction to Measure Whole Brain Atrophy in Relapsing-Remitting Ms. Multiple Sclerosis Collaborative Research Group. *Neurology* (1999) 53(8):1698-704. Epub 1999/11/24. doi: 10.1212/wnl.53.8.1698.

20. Calabresi PA, Kieseier BC, Arnold DL, Balcer LJ, Boyko A, Pelletier J, et al. Pegylated Interferon Beta-1a for Relapsing-Remitting Multiple Sclerosis (Advance): A Randomised, Phase 3, Double-Blind Study. *Lancet Neurol* (2014) 13(7):657-65. Epub 2014/05/06. doi: 10.1016/s1474-4422(14)70068-7.

21. Arnold DL, Calabresi PA, Kieseier BC, Liu S, You X, Fiore D, et al. Peginterferon Beta-1a Improves Mri Measures and Increases the Proportion of Patients with No Evidence of Disease Activity in Relapsing-Remitting Multiple Sclerosis: 2-Year Results from the Advance Randomized Controlled Trial. *BMC Neurol* (2017) 17(1):29. Epub 2017/02/12. doi: 10.1186/s12883-017-0799-0.

22. Penner IK, Stemper B, Calabrese P, Freedman MS, Polman CH, Edan G, et al. Effects of Interferon Beta-1b on Cognitive Performance in Patients with a First Event Suggestive of Multiple Sclerosis. *Mult Scler* (2012) 18(10):1466-71. Epub 2012/04/12. doi: 10.1177/1352458512442438.

23. Jacobs L, O'Malley J, Freeman A, Ekes R. Intrathecal Interferon Reduces Exacerbations of Multiple Sclerosis. *Science* (1981) 214(4524):1026-8. Epub 1981/11/27. doi: 10.1126/science.6171035.

24. Jacobs LD, Cookfair DL, Rudick RA, Herndon RM, Richert JR, Salazar AM, et al. Intramuscular Interferon Beta-1a for Disease Progression in Relapsing Multiple Sclerosis. The Multiple Sclerosis Collaborative Research Group (Mscrg). *Ann Neurol* (1996) 39(3):285-94. Epub 1996/03/01. doi: 10.1002/ana.410390304.

25. Interferon Beta-1b Is Effective in Relapsing-Remitting Multiple Sclerosis. I. Clinical Results of a Multicenter, Randomized, Double-Blind, Placebo-Controlled Trial. The Ifnb Multiple Sclerosis Study Group. *Neurology* (1993) 43(4):655-61. Epub 1993/04/01. doi: 10.1212/wnl.43.4.655.

26. Placebo-Controlled Multicentre Randomised Trial of Interferon Beta-1b in Treatment of Secondary Progressive Multiple Sclerosis. European Study Group on Interferon Beta-1b in Secondary Progressive Ms. *Lancet* (1998) 352(9139):1491-7. Epub 1998/11/20. doi: 10.1212/wnl.43.4.655.

27. Panitch H, Miller A, Paty D, Weinshenker B. Interferon Beta-1b in Secondary Progressive Ms: Results from a 3-Year Controlled Study. *Neurology* (2004) 63(10):1788-95. Epub 2004/11/24. doi: 10.1212/01.wnl.0000146958.77317.3e.

28. Kita M, Goodkin DE, Bacchetti P, Waubant E, Nelson SJ, Majumdar S. Magnetization Transfer Ratio in New Ms Lesions before and During Therapy with Ifnbeta-1a. *Neurology* (2000) 54(9):1741-5. Epub 2000/05/10. doi: 10.1212/wnl.54.9.1741.

29. Aharoni R, Herschkovitz A, Eilam R, Blumberg-Hazan M, Sela M, Bruck W, et al. Demyelination Arrest and Remyelination Induced by Glatiramer Acetate Treatment of Experimental Autoimmune Encephalomyelitis. *Proc Natl Acad Sci U S A* (2008) 105(32):11358-63. Epub 2008/08/06. doi: 10.1073/pnas.0804632105.

30. Skihar V, Silva C, Chojnacki A, Döring A, Stallcup WB, Weiss S, et al. Promoting Oligodendrogenesis and Myelin Repair Using the Multiple Sclerosis Medication Glatiramer Acetate. *Proc Natl Acad Sci U S A* (2009) 106(42):17992-7. Epub 2009/10/10. doi: 10.1073/pnas.0909607106.

31. Babaesfahani A, Bajaj T. Glatiramer. *Statpearls*. Treasure Island, FL: StatPearls Publishing (2022).

32. Comi G, Martinelli V, Rodegher M, Moiola L, Bajenaru O, Carra A, et al. Effect of Glatiramer Acetate on Conversion to Clinically Definite Multiple Sclerosis in Patients with Clinically Isolated Syndrome (Precise Study): A Randomised, Double-Blind, Placebo-Controlled Trial. *Lancet* (2009) 374(9700):1503-11. Epub 2009/10/10. doi: 10.1016/s0140-6736(09)61259-9.

33. Khan O, Rieckmann P, Boyko A, Selmaj K, Zivadinov R. Three Times Weekly Glatiramer Acetate in Relapsing-Remitting Multiple Sclerosis. *Ann Neurol* (2013) 73(6):705-13. Epub 2013/05/21. doi: 10.1002/ana.23938.

34. Weinstein A, Schwid SR, Schiffer RB, McDermott MP, Giang DW, Goodman AD. Neuropsychologic Status in Multiple Sclerosis after Treatment with Glatiramer. *Arch Neurol* (1999) 56(3):319-24. Epub 1999/04/06. doi: 10.1001/archneur.56.3.319.

35. Johnson KP, Brooks BR, Cohen JA, Ford CC, Goldstein J, Lisak RP, et al. Copolymer 1 Reduces Relapse Rate and Improves Disability in Relapsing-Remitting Multiple Sclerosis: Results of a Phase Iii Multicenter, Double-Blind Placebo-Controlled Trial. The Copolymer 1 Multiple Sclerosis Study Group. *Neurology* (1995) 45(7):1268-76. Epub 1995/07/01. doi: 10.1212/wnl.45.7.1268.

36. Brown RA, Narayanan S, Stikov N, Cook S, Cadavid D, Wolansky L, et al. Mtr Recovery in Brain Lesions in the Become Study of Glatiramer Acetate Vs Interferon Β-1b. *Neurology* (2016) 87(9):905-11. Epub 2016/07/31. doi: 10.1212/wnl.0000000000003043.

37. Burns SA, Lee Archer R, Chavis JA, Tull CA, Hensley LL, Drew PD. Mitoxantrone Repression of Astrocyte Activation: Relevance to Multiple Sclerosis. *Brain Res* (2012) 1473:236-41. Epub 2012/08/14. doi: 10.1016/j.brainres.2012.07.054.

38. El-Emam MA, El Achy S, Abdallah DM, El-Abhar HS, Gowayed MA. Does Physical Exercise Improve or Deteriorate Treatment of Multiple Sclerosis with Mitoxantrone? Experimental Autoimmune Encephalomyelitis Study in Rats. *BMC Neurosci* (2022) 23(1):11. Epub 2022/03/07. doi: 10.1186/s12868-022-00692-1.

39. Hensley LL, Burns SA, Chavis JA, Neal JT, Ngwanyam RR, Busby KE, et al. Mitoxantrone Represses Markers of Microglial Activation and Inflammation [Abstract]. *FASEB J* (2006) 20(5):A879. doi: https://doi.org/10.1096/fasebj.20.5.A879-c.

40. Novantrone and Associated Names [Summary of Product Characteristics]: European Medicines Agency (2016) [July 22, 2022]. Available from: https://www.ema.europa.eu/en/documents/referral/novantrone-article-30-referral-annex-iii_en.pdf.

41. Hartung HP, Gonsette R, König N, Kwiecinski H, Guseo A, Morrissey SP, et al. Mitoxantrone in Progressive Multiple Sclerosis: A Placebo-Controlled, Double-Blind, Randomised, Multicentre Trial. *Lancet* (2002) 360(9350):2018-25. Epub 2002/12/31. doi: 10.1016/s0140-6736(02)12023-x.

42. Millefiorini E, Gasperini C, Pozzilli C, D'Andrea F, Bastianello S, Trojano M, et al. Randomized Placebo-Controlled Trial of Mitoxantrone in Relapsing-Remitting Multiple Sclerosis: 24-Month Clinical and Mri Outcome. *J Neurol* (1997) 244(3):153-9. Epub 1997/03/01. doi: 10.1007/s004150050066.

43. Kapoor R, Ho PR, Campbell N, Chang I, Deykin A, Forrestal F, et al. Effect of Natalizumab on Disease Progression in Secondary Progressive Multiple Sclerosis (Ascend): A Phase 3, Randomised, Double-Blind, Placebo-Controlled Trial with an Open-Label Extension. *Lancet Neurol* (2018) 17(5):405-15. Epub 2018/03/17. doi: 10.1016/s1474-4422(18)30069-3.

44. Miller DH, Soon D, Fernando KT, MacManus DG, Barker GJ, Yousry TA, et al. Mri Outcomes in a Placebo-Controlled Trial of Natalizumab in Relapsing Ms. *Neurology* (2007) 68(17):1390-401. doi: 10.1212/01.wnl.0000260064.77700.fd.

45. Weinstock-Guttman B, Galetta SL, Giovannoni G, Havrdova E, Hutchinson M, Kappos L, et al. Additional Efficacy Endpoints from Pivotal Natalizumab Trials in Relapsing-Remitting Ms. *J Neurol* (2012) 259(5):898-905. doi: 10.1007/s00415-011-6275-7.

46. Polman CH, O'Connor PW, Havrdova E, Hutchinson M, Kappos L, Miller DH, et al. A Randomized, Placebo-Controlled Trial of Natalizumab for Relapsing Multiple Sclerosis. *N Engl J Med* (2006) 354(9):899-910. doi: 10.1056/NEJMoa044397.

47. Zivadinov R, Dwyer MG, Hussein S, Carl E, Kennedy C, Andrews M, et al. Voxel-Wise Magnetization Transfer Imaging Study of Effects of Natalizumab and Ifn Beta-1a in Multiple Sclerosis. *Mult Scler* (2012) 18(8):1125-34. doi: 10.1177/1352458511433304.

48. Hauser SL, Bar-Or A, Comi G, Giovannoni G, Hartung HP, Hemmer B, et al. Ocrelizumab Versus Interferon Beta-1a in Relapsing Multiple Sclerosis. *N Engl J Med* (2017) 376(3):221-34. Epub 2016/12/22. doi: 10.1056/NEJMoa1601277.

49. Montalban X, Hauser SL, Kappos L, Arnold DL, Bar-Or A, Comi G, et al. Ocrelizumab Versus Placebo in Primary Progressive Multiple Sclerosis. *N Engl J Med* (2017) 376(3):209-20. Epub 2016/12/22. doi: 10.1056/NEJMoa1606468.

50. Benedict RHB, de Seze J, Hauser SL, Kappos L, Wolinsky JS, Zheng H, et al., editors. Impact of Ocrelizumab on Cognition in Patients at Increased Risk of Progressive Disease [Poster]. *Annual Meeting of the American Academy of Neurology*; 2018 April 21-27, 2018; Los Angeles, CA.

51. Kolind S, Abel S, Taylor C, Tam R, Laule C, Li DKB, et al. Myelin Water Imaging in Relapsing Multiple Sclerosis Treated with Ocrelizumab and Interferon Beta-1a. *Neuroimage Clin* (2022) 35:103109. Epub 2022/07/26. doi: 10.1016/j.nicl.2022.103109.

52. Hauser SL, Bar-Or A, Cohen JA, Comi G, Correale J, Coyle PK, et al. Ofatumumab Versus Teriflunomide in Multiple Sclerosis. *N Engl J Med* (2020) 383(6):546-57. Epub 2020/08/07. doi: 10.1056/NEJMoa1917246.

53. Benedict RHB, Penner IK, Cutter G, Kappos L, Coyle PK, Piani-Meier D, et al. Improvement in Cognitive Processing Speed with Ofatumumab in Patients with Relapsing Multiple Sclerosis [Abstract Opr-130]. *Eur J Neurol* (2022) 29(suppl 1):149.

54. Göttle P, Manousi A, Kremer D, Reiche L, Hartung HP, Küry P. Teriflunomide Promotes Oligodendroglial Differentiation and Myelination. *J Neuroinflammation* (2018) 15(1):76. Epub 2018/03/15. doi: 10.1186/s12974-018-1110-z.

55. Kabiraj P, Grund EM, Clarkson BDS, Johnson RK, LaFrance-Corey RG, Lucchinetti CF, et al. Teriflunomide Shifts the Astrocytic Bioenergetic Profile from Oxidative Metabolism to Glycolysis and Attenuates Tnfα-Induced Inflammatory Responses. *Sci Rep* (2022) 12(1):3049. Epub 2022/02/25. doi: 10.1038/s41598-022-07024-7.

56. Martin E, Aigrot MS, Lamari F, Bachelin C, Lubetzki C, Nait Oumesmar B, et al. Teriflunomide Promotes Oligodendroglial 8,9-Unsaturated Aterol Accumulation and Cns Remyelination. *Neurol Neuroimmunol Neuroinflamm* (2021) 8(6). Epub 2021/10/14. doi: 10.1212/nxi.0000000000001091.

57. Wostradowski T, Prajeeth CK, Gudi V, Kronenberg J, Witte S, Brieskorn M, et al. In Vitro Evaluation of Physiologically Relevant Concentrations of Teriflunomide on Activation and Proliferation of Primary Rodent Microglia. *J Neuroinflammation* (2016) 13(1):250. Epub 2016/09/24. doi: 10.1186/s12974-016-0715-3.

58. Radue EW, Sprenger T, Gaetano L, Mueller-Lenke N, Cavalier S, Thangavelu K, et al. Teriflunomide Slows Bvl in Relapsing Ms: A Reanalysis of the Temso Mri Data Set Using Siena. *Neurol Neuroimmunol Neuroinflamm* (2017) 4(5):e390. Epub 2017/08/23. doi: 10.1212/nxi.0000000000000390.

59. Sprenger T, Kappos L, Sormani MP, Miller AE, Poole EM, Cavalier S, et al. Effects of Teriflunomide Treatment on Cognitive Performance and Brain Volume in Patients with Relapsing Multiple Sclerosis: Post Hoc Analysis of the Temso Core and Extension Studies. *Mult Scler* (2022) 28(11):1719-28. Epub 2022/04/30. doi: 10.1177/13524585221089534.

60. Confavreux C, O'Connor P, Comi G, Freedman MS, Miller AE, Olsson TP, et al. Oral Teriflunomide for Patients with Relapsing Multiple Sclerosis (Tower): A Randomised, Double-Blind, Placebo-Controlled, Phase 3 Trial. *Lancet Neurol* (2014) 13(3):247-56. Epub 2014/01/28. doi: 10.1016/s1474-4422(13)70308-9.

61. O'Connor P, Wolinsky JS, Confavreux C, Comi G, Kappos L, Olsson TP, et al. Randomized Trial of Oral Teriflunomide for Relapsing Multiple Sclerosis. *N Engl J Med* (2011) 365(14):1293-303. doi: 10.1056/NEJMoa1014656.

62. Berger AA, Sottosanti ER, Winnick A, Izygon J, Berardino K, Cornett EM, et al. Monomethyl Fumarate (Mmf, Bafiertam) for the Treatment of Relapsing Forms of Multiple Sclerosis (Ms). *Neurol Int* (2021) 13(2):207-23. Epub 2021/06/03. doi: 10.3390/neurolint13020022.

63. Naismith RT, Wolinsky JS, Wundes A, LaGanke C, Arnold DL, Obradovic D, et al. Diroximel Fumarate (Drf) in Patients with Relapsing-Remitting Multiple Sclerosis: Interim Safety and Efficacy Results from the Phase 3 Evolve-Ms-1 Study. *Mult Scler* (2020) 26(13):1729-39. Epub 2019/11/05. doi: 10.1177/1352458519881761.

64. Naismith RT, Wundes A, Ziemssen T, Jasinska E, Freedman MS, Lembo AJ, et al. Diroximel Fumarate Demonstrates an Improved Gastrointestinal Tolerability Profile Compared with Dimethyl Fumarate in Patients with Relapsing-Remitting Multiple Sclerosis: Results from the Randomized, Double-Blind,Phase Iii Evolve-Ms-2 Study. *CNS Drugs* (2020) 34(2):185-96. Epub 2020/01/19. doi: 10.1007/s40263-020-00700-0.

65. Wray S, Then Bergh F, Wundes A, Arnold DL, Drulovic J, Jasinska E, et al. Efficacy and Safety Uutcomes with Diroximel Fumarate after Switching from Prior Therapies or Continuing on Drf: Results from the Phase 3 Evolve-Ms-1 Study. *Adv Ther* (2022) 39(4):1810-31. Epub 2022/02/26. doi: 10.1007/s12325-022-02068-7.

66. Jørgensen L, Hyrlov KH, Elkjaer ML, Weber AB, Pedersen AE, Svenningsen Å F, et al. Cladribine Modifies Functional Properties of Microglia. *Clin Exp Immunol* (2020) 201(3):328-40. Epub 2020/06/04. doi: 10.1111/cei.13473.

67. Singh V, Voss EV, Bénardais K, Stangel M. Effects of 2-Chlorodeoxyadenosine (Cladribine) on Primary Rat Microglia. *J Neuroimmune Pharmacol* (2012) 7(4):939-50. Epub 2012/07/24. doi: 10.1007/s11481-012-9387-7.

68. Aybar F, Julia Perez M, Silvina Marcora M, Eugenia Samman M, Marrodan M, María Pasquini J, et al. 2-Chlorodeoxyadenosine (Cladribine) Preferentially Inhibits the Biological Activity of Microglial Cells. *Int Immunopharmacol* (2022) 105:108571. Epub 2022/01/31. doi: 10.1016/j.intimp.2022.108571.

69. Kearns CM, Blakley RL, Santana VM, Crom WR. Pharmacokinetics of Cladribine (2-Chlorodeoxyadenosine) in Children with Acute Leukemia. *Cancer Res* (1994) 54(5):1235-9. Epub 1994/03/01.

70. De Stefano N, Giorgio A, Battaglini M, De Leucio A, Hicking C, Dangond F, et al. Reduced Brain Atrophy Rates Are Associated with Lower Risk of Disability Progression in Patients with Relapsing Multiple Sclerosis Treated with Cladribine Tablets. *Mult Scler* (2018) 24(2):222-6. Epub 2017/02/01. doi: 10.1177/1352458517690269.

71. Giovannoni G, Comi G, Cook S, Rammohan K, Rieckmann P, Soelberg Sørensen P, et al. A Placebo-Controlled Trial of Oral Cladribine for Relapsing Multiple Sclerosis. *N Engl J Med* (2010) 362(5):416-26. Epub 2010/01/22. doi: 10.1056/NEJMoa0902533.

72. Choi JW, Gardell SE, Herr DR, Rivera R, Lee CW, Noguchi K, et al. Fty720 (Fingolimod) Efficacy in an Animal Model of Multiple Sclerosis Requires Astrocyte Sphingosine 1-Phosphate Receptor 1 (S1p1) Modulation. *Proc Natl Acad Sci U S A* (2011) 108(2):751-6. Epub 2010/12/24. doi: 10.1073/pnas.1014154108.

73. Zhang J, Zhang ZG, Li Y, Ding X, Shang X, Lu M, et al. Fingolimod Treatment Promotes Proliferation and Differentiation of Oligodendrocyte Progenitor Cells in Mice with Experimental Autoimmune Encephalomyelitis. *Neurobiol Dis* (2015) 76:57-66. Epub 2015/02/15. doi: 10.1016/j.nbd.2015.01.006.

74. Jackson SJ, Giovannoni G, Baker D. Fingolimod Modulates Microglial Activation to Augment Markers of Remyelination. *J Neuroinflammation* (2011) 8:76. Epub 2011/07/07. doi: 10.1186/1742-2094-8-76.

75. Doi Y, Takeuchi H, Horiuchi H, Hanyu T, Kawanokuchi J, Jin S, et al. Fingolimod Phosphate Attenuates Oligomeric Amyloid Β-Induced Neurotoxicity Via Increased Brain-Derived Neurotrophic Factor Expression in Neurons. *PLoS One* (2013) 8(4):e61988. Epub 2013/04/18. doi: 10.1371/journal.pone.0061988.

76. Foster CA, Howard LM, Schweitzer A, Persohn E, Hiestand PC, Balatoni B, et al. Brain Penetration of the Oral Immunomodulatory Drug Fty720 and Its Phosphorylation in the Central Nervous System During Experimental Autoimmune Encephalomyelitis: Consequences for Mode of Action in Multiple Sclerosis. *J Pharmacol Exp Ther* (2007) 323(2):469-75. Epub 2007/08/08. doi: 10.1124/jpet.107.127183.

77. Tamagnan G, Tavares A, Barret O, Alagille D, Seibyl J, Marek K, et al. Brain Distribution of Bzm055, an Analog of Fingolimod (Fty720), in Human [Abstract P839]. *Mult Scler* (2012) 18(suppl 4).

78. Kappos L, Radue EW, O'Connor P, Polman C, Hohlfeld R, Calabresi P, et al. A Placebo-Controlled Trial of Oral Fingolimod in Relapsing Multiple Sclerosis. *N Engl J Med* (2010) 362(5):387-401. Epub 2010/01/22. doi: 10.1056/NEJMoa0909494.

79. Cohen JA, Barkhof F, Comi G, Hartung HP, Khatri BO, Montalban X, et al. Oral Fingolimod or Intramuscular Interferon for Relapsing Multiple Sclerosis. *N Engl J Med* (2010) 362(5):402-15. Epub 2010/01/22. doi: 10.1056/NEJMoa0907839.

80. Calabresi PA, Radue EW, Goodin D, Jeffery D, Rammohan KW, Reder AT, et al. Safety and Efficacy of Fingolimod in Patients with Relapsing-Remitting Multiple Sclerosis (Freedoms Ii): A Double-Blind, Randomised, Placebo-Controlled, Phase 3 Trial. *Lancet Neurol* (2014) 13(6):545-56. Epub 2014/04/02. doi: 10.1016/s1474-4422(14)70049-3.

81. Langdon DW, Tomic D, Penner IK, Calabrese P, Cutter G, Häring DA, et al. Baseline Characteristics and Effects of Fingolimod on Cognitive Performance in Patients with Relapsing-Remitting Multiple Sclerosis. *Eur J Neurol* (2021) 28(12):4135-45. Epub 2021/08/26. doi: 10.1111/ene.15081.

82. Preziosa P, Storelli L, Meani A, Moiola L, Rodegher M, Filippi M, et al. Effects of Fingolimod and Natalizumab on Brain T1-/T2-Weighted and Magnetization Transfer Ratios: A 2-Year Study. *Neurotherapeutics* (2021) 18(2):878-88. Epub 2021/01/24. doi: 10.1007/s13311-020-00997-1.

83. Scott FL, Clemons B, Brooks J, Brahmachary E, Powell R, Dedman H, et al. Ozanimod (Rpc1063) Is a Potent Sphingosine-1-Phosphate Receptor-1 (S1p1 ) and Receptor-5 (S1p5 ) Agonist with Autoimmune Disease-Modifying Activity. *Br J Pharmacol* (2016) 173(11):1778-92. Epub 2016/03/19. doi: 10.1111/bph.13476.

84. Taylor Meadows KR, Selkirk JV, Akhtar MW, Hutton C, Opiteck GJ, Scott FL. Ozanimod (Rpc1063) Is Potentially Neuroprotective through Direct Cns Effects [Abstract P1183]. *Mult Scler* (2017) 23(suppl 3):624.

85. Musella A, Gentile A, Guadalupi L, Rizzo FR, De Vito F, Fresegna D, et al. Central Modulation of Selective Sphingosine-1-Phosphate Receptor 1 Ameliorates Experimental Multiple Sclerosis. *Cells* (2020) 9(5). Epub 2020/05/28. doi: 10.3390/cells9051290.

86. Selkirk JV, Yan YG, Ching N, Paget K, Hargreaves R. In Vitro Assessment of the Binding and Functional Reponses of Ozanimod and Its Plasma Metabolites across Human Sphingosine 1-Phosphate Receptors. *Eur J Pharmacol* (2022):175442. Epub 2022/12/06. doi: 10.1016/j.ejphar.2022.175442.

87. Cohen JA, Comi G, Selmaj KW, Bar-Or A, Arnold DL, Steinman L, et al. Safety and Efficacy of Ozanimod Versus Interferon Beta-1a in Relapsing Multiple Sclerosis (Radiance): A Multicentre, Randomised, 24-Month, Phase 3 Trial. *Lancet Neurol* (2019) 18(11):1021-33. doi: 10.1016/S1474-4422(19)30238-8.

88. Comi G, Kappos L, Selmaj KW, Bar-Or A, Arnold DL, Steinman L, et al. Safety and Efficacy of Ozanimod Versus Interferon Beta-1a in Relapsing Multiple Sclerosis (Sunbeam): A Multicentre, Randomised, Minimum 12-Month, Phase 3 Trial. *Lancet Neurol* (2019) 18(11):1009-20. doi: 10.1016/S1474-4422(19)30239-X.

89. Cree BA, Selmaj KW, Steinman L, Comi G, Bar-Or A, Arnold DL, et al. Long-Term Safety and Efficacy of Ozanimod in Relapsing Multiple Sclerosis: Up to 5 Years of Follow-up in the Daybreak Open-Label Extension Trial. *Mult Scler* (2022) 28 (12):1944-62. doi: 10.1177/13524585221102584.

90. Fourgeaud L, Le M, Needham A, Ait-Tihyaty M, Lair LL, Breu V, et al. A Central Effect of Ponesimod on Neuroinflammation in a Pre-Clinical Model of Multiple Sclerosis [Abstract P191]. *Mult Scler* (2021) 27(1 suppl):105.

91. Kihara Y, Jonnalagadda D, Zhu Y, Ray M, Ngo T, Palmer C, et al. Ponesimod Inhibits Astrocyte-Mediated Neuroinflammation and Protects against Cingulum Demyelination Via S1p(1) -Selective Modulation. *FASEB J* (2022) 36(2):e22132. Epub 2022/01/06. doi: 10.1096/fj.202101531R.

92. Kappos L, Fox RJ, Burcklen M, Freedman MS, Havrdová EK, Hennessy B, et al. Ponesimod Compared with Teriflunomide in Patients with Relapsing Multiplesclerosis in the Active-Comparator Phase 3 Optimum Study: A Randomized Clinical Trial. *JAMA Neurol* (2021) 78(5):558-67. Epub 2021/03/30. doi: 10.1001/jamaneurol.2021.0405.

93. Fox R, Kappos L, Burcklen M, Freedman M, Havrdova E, Hennessy B, et al. Effect on Disability Measures and Msfc in Patients with Relapsing Multiple Sclerosis from the Phase 3 Ponesimod Versus Teriflunomide Optimum Study [Abstract P0204]. *Mult Scler* (2020) 26(suppl 3):216-7.

94. Tiwari-Woodruff S, Yamate-Morgan H, Sekyi M, Lauderdale K, Hasselmann J, Schubart A. The Sphingosine 1-Phosphate (S1p) Receptor Modulator, Siponimod Decreases Oligodendrocyte Cell Death and Axon Demyelination in a Mouse Model of Multiple Sclerosis [Abstract]. *Neurology* (2016) 86(16 suppl):I10.011.

95. Mannioui A, Vauzanges Q, Fini JB, Henriet E, Sekizar S, Azoyan L, et al. The Xenopus Tadpole: An in Vivo Model to Screen Drugs Favoring Remyelination. *Mult Scler* (2018) 24(11):1421-32. Epub 2017/07/29. doi: 10.1177/1352458517721355.

96. Colombo E, Bassani C, De Angelis A, Ruffini F, Ottoboni L, Comi G, et al. Siponimod (Baf312) Activates Nrf2 While Hampering Nfκb in Human Astrocytes, and Protects from Astrocyte-Induced Neurodegeneration. *Front Immunol* (2020) 11:635. Epub 2020/04/24. doi: 10.3389/fimmu.2020.00635.

97. Gentile A, Musella A, Bullitta S, Fresegna D, De Vito F, Fantozzi R, et al. Siponimod (Baf312) Prevents Synaptic Neurodegeneration in Experimental Multiple Sclerosis. *J Neuroinflammation* (2016) 13(1):207. Epub 2016/08/28. doi: 10.1186/s12974-016-0686-4.

98. Hundehege P, Cerina M, Eichler S, Thomas C, Herrmann AM, Göbel K, et al. The Next-Generation Sphingosine-1 Receptor Modulator Baf312 (Siponimod) Improves Cortical Network Functionality in Focal Autoimmune Encephalomyelitis. *Neural Regen Res* (2019) 14(11):1950-60. Epub 2019/07/11. doi: 10.4103/1673-5374.259622.

99. Dietrich M, Hecker C, Martin E, Langui D, Gliem M, Stankoff B, et al. Increased Remyelination and Proregenerative Microglia under Siponimod Therapy in Mechanistic Models. *Neurol Neuroimmunol Neuroinflamm* (2022) 9(3). Epub 2022/04/01. doi: 10.1212/nxi.0000000000001161.

100. Bigaud M, Rudolph B, Briard E, Beerli C, Schubart A, Gardin A. Siponimod Penetrates, Distributes and Acts on the Central Nervous System: Translational Insights [Abstract]. *Neurology* (2020) 94(15 suppl):3973.

101. Kappos L, Bar-Or A, Cree BAC, Fox RJ, Giovannoni G, Gold R, et al. Siponimod Versus Placebo in Secondary Progressive Multiple Sclerosis (Expand): A Double-Blind, Randomised, Phase 3 Study. *Lancet* (2018) 391(10127):1263-73. Epub 2018/03/27. doi: 10.1016/s0140-6736(18)30475-6.

102. Benedict RHB, Tomic D, Fox R, Cree BAC, Vermersch P, Giovannoni G, et al., editors. Siponimod Improves Cognitive Processing Speed in Patients with Secondary Progressive Multiple Sclerosis: Expand Subgroup Analyses [Poster P22]. *Annual meeting of the International MS Cognition Society*; 2019 June 6-7, 2019; Amsterdam, Netherlands.

103. Arnold DL, Vermersch P, Cree BAC, Bar-or A, Giovannoni G, Gold R, et al. Evidence for Improved Myelination in Patients Treated with Siponimod: Results from the Phase 3 Expand Mri Substudy [Abstract Epr1147]. *Eur J Neurol* (2020) 27(suppl 1):194-5.

104. Arnold DL, Piani-Meier D, Bar-Or A, Benedict RH, Cree BA, Giovannoni G, et al. Effect of Siponimod on Magnetic Resonance Imaging Measures of Neurodegeneration and Myelination in Secondary Progressive Multiple Sclerosis: Gray Matter Atrophy and Magnetization Transfer Ratio Analyses from the Expand Phase 3 Trial. *Mult Scler* (2022) 28(10):1526-40. Epub 2022/03/10. doi: 10.1177/13524585221076717.
